# Frame-shifted proteins of a given gene retain the same function

**DOI:** 10.1093/nar/gkaa169

**Published:** 2020-03-18

**Authors:** Xin Huang, Rong Chen, Meiling Sun, Yan Peng, Qinlin Pu, Yi Yuan, Gangyi Chen, Juan Dong, Feng Du, Xin Cui, Zhuo Tang

**Affiliations:** 1 Natural Products Research Center, Chengdu Institution of Biology, Chinese Academy of Science, Chengdu 610041, P. R. China; 2 Ethnomedicine College, Chengdu University of Traditional Chinese Medicine, Chengdu 610041, P. R. China

## Abstract

Frameshift mutations are generally considered to be lethal because it could result in radical changes of the protein sequence behind. However, the protein of frameshift mutants of a type I toxin (*ibsc*) was found to be still toxic to bacteria, retaining the similar function as wild-type protein to arrest the cellular growth by impairing the membrane's integrity. Additionally, we have verified that this observation is not an individual event as the same phenomenon had been found in other toxins subsequently. After analyzing the coding sequence of these genes, we proposed a hypothesis to search this kind of hidden gene, through which a dihydrofolate reductase-encoding gene (*dfrB3*) was found out. Like the wild-type reductase, both +1 and –1 frame-shifted proteins of *dfrB3* gene were also proved to catalyze the reduction of dihydrofolate to tetrahydrofolate by using NADPH.

## INTRODUCTION

**Figure 1.**
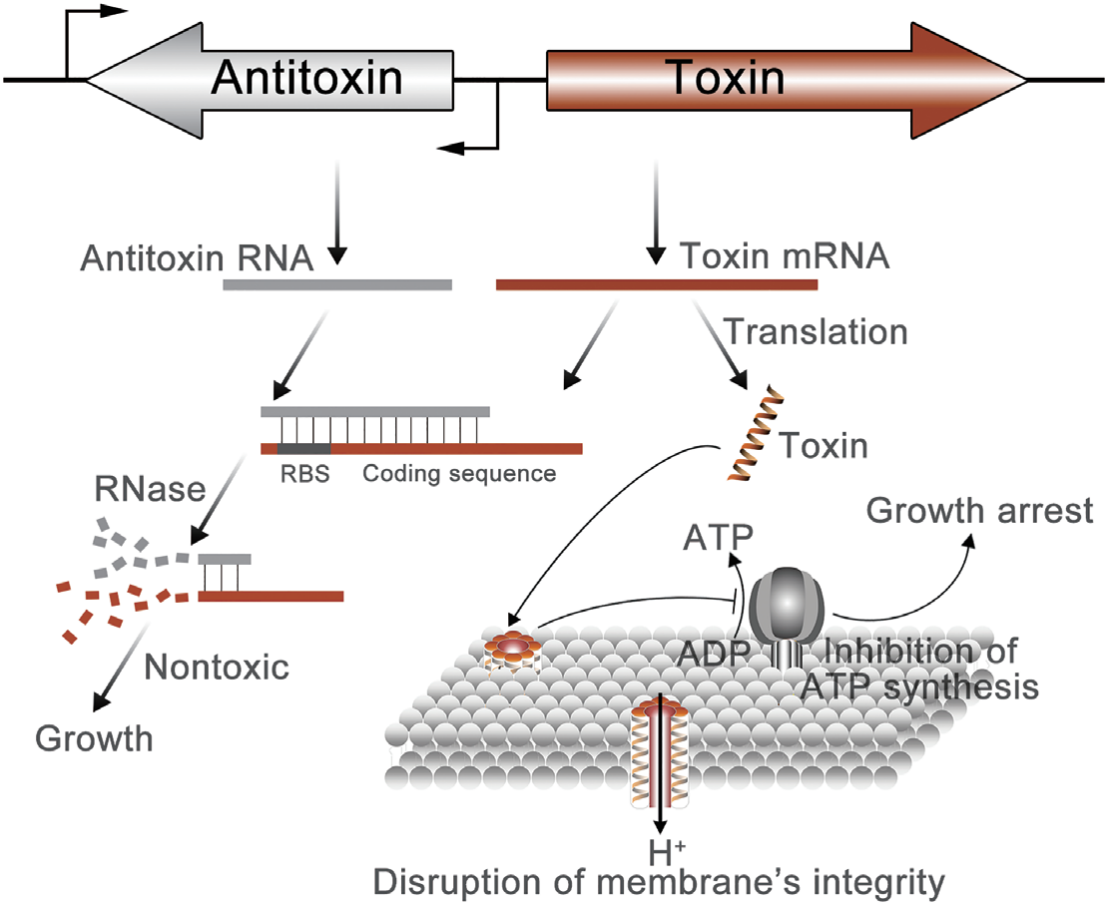
Type I toxin–antitoxin system. Toxin and RNA–antitoxin are transcribed separately, and antitoxin RNA binds to mRNAs of toxin to form a duplex, thus disrupting the translation through the inhibition of ribosome binding or RNA degradation by RNase. Type I toxins have an identical α-helix structure to form a pore across the inner membrane to impair the transmembrane proton gradient to inhibit ATP synthesis and cause the growth arrest.

The toxin and antitoxin systems (TA systems) are small genetic elements, which are widely prevalent in plasmids, phages and on chromosomes of most free-living bacteria. They are composed of a toxin, which leads to growth arrest by interfering with a vital cellular process, and a cognate antitoxin, which counteracts the toxin activity (1). Currently, TA systems are assigned to six classes (I–VI) according to their regulation mechanisms. Toxins are proteins in all identified bacterial TA systems, while antitoxins are either small RNAs (in Type I and III) or proteins (in Type II, IV, V and VI). Type I TA system was initially discovered in bacterial plasmid as a post-segregational killing system (2). Indeed, all Type I toxins share a common secondary structure, α-helix, to form a pore across the inner membrane to impair the transmembrane proton gradient to inhibit ATP synthesis (Figure 1) (3–7). In Type I TA system, the expression of toxin is regulated by an antisense RNA transcribed from the reverse orientation of toxin gene. The antisense RNA antitoxins can anneal to the toxin mRNAs to form a double-stranded RNA molecule, thus neutralizing toxin activity through inhibition of ribosome binding or RNA degradation by RNase III. In type I TA loci, the hok-sok system is the first and best-characterized locus identified on R1 plasmid of *Escherichia coli*, which contributes to plasmid stability in several gram-negative bacteria (8).

Various bacteria contain unequal TA operons. Usually, bacteria living in constant environments, such as obligate host-associated organisms, tend to exclude TA loci. However, free-living bacteria characterized by very low growth rates in changing environments, contain more TA loci. For instance, pathogenic *Mycobacterium tuberculosis* has more than 80 TA systems (9), while a fast-growing *Mycobacterium smegmatis* has only two TA systems (10). It has been speculated that the TA systems may help the *M. tuberculosis* to maintain the extremely long dormancy in host cells. These extensive TA loci systems are tightly associated with bacterial persistence, the high tolerant status for nutrient starvation or antibiotic exposure. Under stressed conditions, the antitoxins of bacteria are selectively degraded, thus leaving toxins to inhibit cell growth. Once the environment stresses are removed, the bacteria will return to the normal growth pattern. Therefore, TA systems are essential for the survival and evolution of bacteria.

As one type of gene mutation, the frameshift is defined as protein translations that start not at the first, but at the second (+1 frameshift or **+1 fs**) or the third (–1 frameshift or **–1 fs**) of the codon. Due to the triplet nature of codons, the amino acid sequence of a protein is determined by contiguous triplets. Therefore, a frameshift mutation leads to completely different proteins in term of chemical composition, and the closer to 5′ end of coding sequence the mutation occurs, the more protein changes it can make. As gene frameshift would yield nonfunctional or cytotoxic proteins, to avoid the negative effect of frameshift organisms have evolved a mechanism to terminate frame-shifted translation by using off-frame stops (stops in +1 and -1 shifted reading frames, termed hidden stops) (11). When we were studying a type I toxin, termed *ibsC*, it was surprisingly found that both **+1 fs** and **–1 fs** mutants of this toxin still retain toxicity to bacteria. After verifying the validity of the results, we found the same phenomenon in other proteins as well, which implies the function of certain genes could be accomplished not only by the apparent encoded protein but also by its hidden frame-shifted proteins.

## MATERIALS AND METHODS

### Bacterial strains and plasmid constructions

The *E. coli* strains used in this study were DH5α and BL21(DE3), purchased from TransGen Biotech (Beijing, China). The investigated genes and its mutants were clone into a modified pET32a vector at restriction endonuclease site BamHI and BlpI. The fusion tags of pET32a was removed and BamHI site, and start codon was re-inserted after RBS of pET32. DNA sequence of investigated genes and related mutants were amplified by primers shown in Supplementary Table S1, and then were purified by a PCR clean kit. The purified DNA fragments and modified pET32a were digested with BamHI and BlpI (NEB, USA) for 3 h at 37°C. Then, the purified inserts and vector DNA were ligated by T4 ligase (NEB, USA) according to the manufacturer's manual. Ligation products were transformed into DH5α chemically competent cell. Inserted sequences on plasmid were confirmed by DNA sequencing.

### Toxicity assays on agar plates

The toxicity of toxin genes and frameshift mutants were evaluated by their effects on the cell growth of *E. coli* on the Luria-Burtani (LB) agar plate. Equal volume of BL21(DE3) cells transformed by recombinants were plated on LB agar with or without IPTG, following incubation at 37°C overnight. One hundred percent toxicity was defined as no one colonies formed on culture plate when mutant protein was overexpressed. Relative toxicity = [1 – (counts on LB agar with IPTG/counts on LB agar without IPTG)] × 100%. These assays have been repeated at least three times.

### Growth curves in liquid medium

Growth curves of transformed cells were used to assess the toxicity or resistance of overexpressed genes and its mutations. BL21(DE3) cells containing WT IbsC and mutants were plated on LB agar for 18 h. At least three independent single bacterial colonies were incubated in 5 ml LB overnight and then diluted 1:200 in fresh medium. Newly inoculated bacteria were incubated in LB medium supplemented with 100 μg ml^−1^ of ampicillin at 37°C with agitation at 250 rpm. For the growth curves of bacteria with toxin genes, IPTG (final concentration of 1 mM) was added when the OD_600_ was 0.1; For the growth curves of *dfrB3* gene, after incubation 1 h at 37°C, IPTG (final concentration of 1 mM) was added to test the resistance to TMP (final concentration of 30 μg/ml). Optical density was followed at 1 h intervals. These assays were repeated at least three times.

### Detection of membrane integrity of Bacteria

BL21(DE3) cells transformed by recombinant vectors were grown overnight and then diluted 1:200 in fresh medium to an OD_600_ of 0.1 in LB. The cells were induced by IPTG to express the corresponding toxin proteins. After 3 h induction, all cells were harvested and stained with DiBAC4(3) with a final concentration of 10 μg/ml, following 20 min incubation at 37°C in the dark. Then cells were pelleted at 4500 rpm for 5 min and washed with phosphate-buffered saline (PBS). The fluorescent intensity of cells was analyzed by flow cytometry in a Moflo DXP (BeckmanCoulter) (excitation at 488 ± 5 nm; emission at 516 ± 5 nm). All parameters were collected as logarithmic signals. The assay was repeated at least three times.

### Substitution of stop codons (TAG or TGA) in frameshift mutant with the code of amino acid

To investigate which amino acids could be inserted in the stop codons (TAG or TGA) in frameshift mutant to retain its function, the stop codons was changed into random sequence NNN by using synthetic primers (Supplementary Table S1). Full length of **+1 fs** of *ibsC* was produced by primers containing random sequence. And full length of **+1 fs** and **–1 fs** of *dfrB3* was prepared using overlap PCR to conjugate three DNA fragments. To cover all potential combinations of AAR substitutions in two stop codons, thousands of clones were picked up and confirmed by DNA sequencing technology, then the toxicity or resistance to antibiotics were tested on agar plate.

### Expression and purification of proteins

Protein sequences of investigated genes for purification were shown in Supplementary Table S2. BL21(DE3) cells transformed by recombinant vectors were grown overnight and diluted 1:200 in fresh medium to OD_600_ 0.4 in LB (250 rpm at 37°C). At this point, the temperature was lowered to 24°C and IPTG (20 μM) was added to induce the expression of DfrB3. The cells were then recovered by centrifugation (8000 rpm at 16°C for 15 min) and resuspended in 50 mM Tris–HCl buffer (pH 7.9) containing 500 mM NaCl and 10 mM imidazole supplemented by 1 mM phenylmethanesulphonylfluoride (PMSF). After mechanical lysis by sonication in an ice-cold bath, the soluble lysate was recovered by centrifugation (8000 rpm at 4°C for 30 min). Then, the soluble fraction was filtered (0.45 μm) and loaded on His column according to the manufactory’ manual. The loaded column was washed with 4 column volumes (CV) of wash buffer (50 mM Tris–HCl buffer pH 7.9 containing 500 mM NaCl and 50 mM imidazole). Finally, the proteins were eluted with 10 ml of elution buffer (50 mM Tris–HCl buffer pH 7.9 containing 500 mM NaCl and 500 mM imidazole) and the imidazole was removed by dialysis membrane. The concentration of purified proteins was measured using nanodrop, and proteins were analyzed with 12% polyacrylamide gel electrophoresis (SDS-PAGE).

### Analysis of the reduction reactions of dihydrofolate with UPLC-MS

Reduction reactions were performed in the presence of DHFA (50 μM), NADPH (100 μM), and DfrB3 proteins (6 μg WT, or 6 μg **+1 fs** (PK), or 12 μg **–1 fs** (GG)) in 50 mM Tris buffer (pH 7.0) and 10 mM β-mercaptoethanol. And 6 μg BSA was applied as the negative control to catalyze the same reduction reaction. All reactions were carried out for 60 min at 37°C, and then stopped by equal volume of methyl alcohol. The reaction mixture was centrifuged at 12 000 rpm for 10 min, and 1 μl of the supernatant was analyzed by UPLC-MS, using a Waters Vion IMS QTof coupled to a Waters Acquity UPLC system. A C18 reverse phase column was held at 40°C and a solvent system of aqueous formic acid 0.1% (v/v) (A) and acetonitrile (B) delivered at a flow rate of 0.2 ml/min. The gradient elution was applied as follows: 0–6 min, 5–25% B; 6–7 min, 25–95% B; 7–10 min, re-equilibration to initial conditions. The reactions and analysis were performed in duplicate. All processing was performed using MassLynx version 4.1. All data were acquired and analyzed by using Masslynx 4.1 software (Waters Corp., Beverly, MA, USA).

## RESULTS

### Validation of toxicity from the frame-shifted protein of *ibsC*

**Figure 2.**
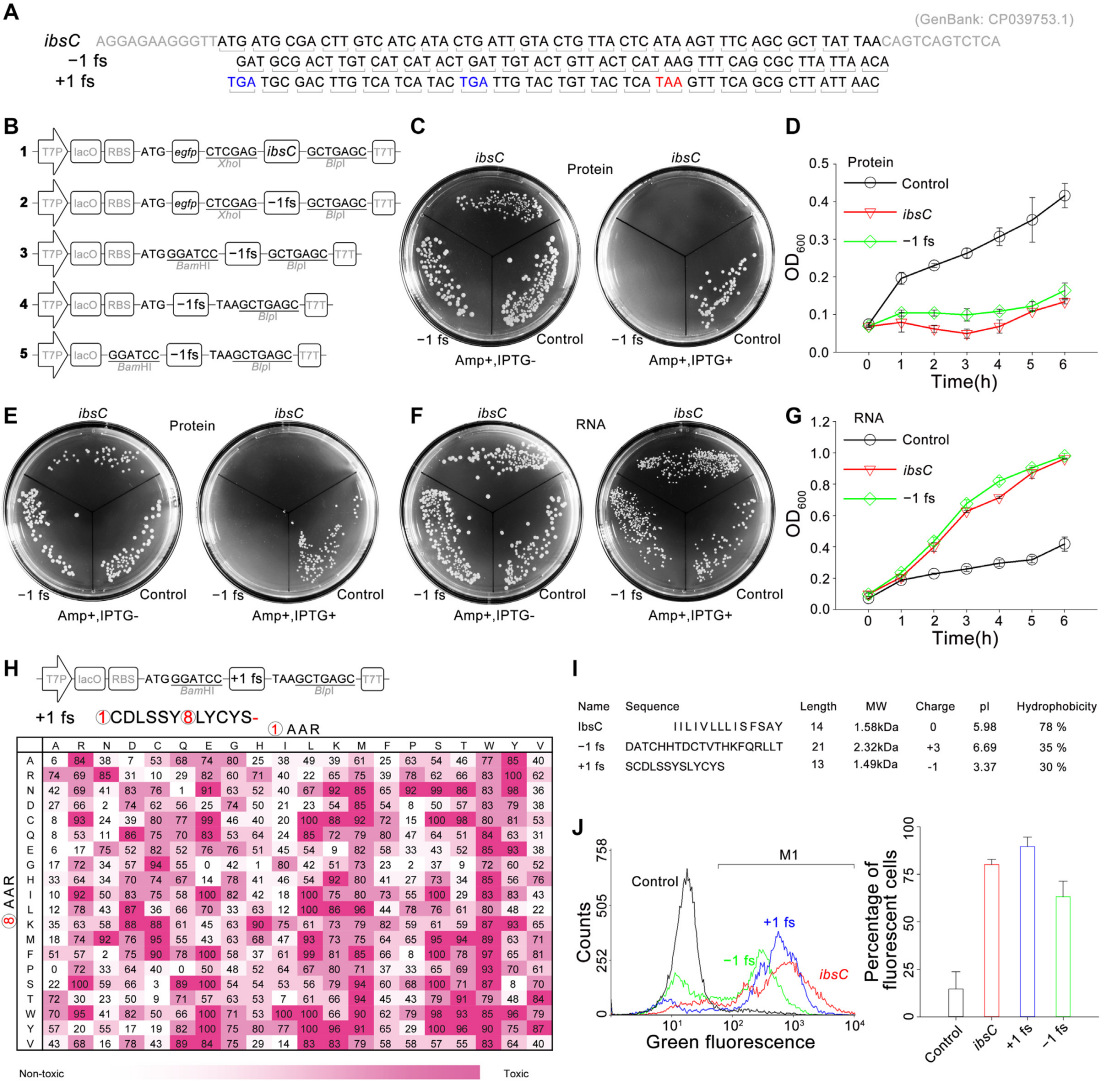
Confirmation of the cellular toxicity of frame-shifted protein of *ibsC*: (**A**) DNA sequence alignment of *ibsC* gene as well as its **–1 fs** and **+1 fs** mutants; (**B**) scheme illustration of expression vectors: **1** (WT IbsC is fusion expressed with EGFP), **2** (**–1 fs** of IbsC is fusion expressed with EGFP), **3** (**–1 fs** is expressed independently), **4** (**–1 fs** is reconstructed by removing the *Bam*HI site and inserting a TAA stop codon), **5** (**–1 fs** without RBS and start codon); (**C**) toxicities of fusion expressed WT and **–1 fs** IbsC on agar plates. Control: *E. coli* with empty vector; (**D**) growth curve lines of *E. coli* containing fusion expressed WT and **–1 fs** proteins of IbsC in liquid LB medium; (**E**) toxicities of WT and **–1 fs** of IbsC constructed on vector **4**; (**F**) toxicities of the RNA of IbsC (WT and **–1 fs**) on agar plate; (**G**) growth curve lines of *E. coli* containing the RNA of IbsC (WT and **–1 fs**) in liquid LB medium; (**H**) systematical substitutions of two predicted stop codons in **+1 fs** by the codes of 20 amino acid, Relative toxicity was tested on agar plate, relative toxicity = (100 – (counts on LB agar with IPTG/counts on LB agar without IPTG) × 100); (**I**) comparison for sequence, length, molecule weight (MW), net charge, isoelectric point (p*I*) and hydrophobicity of active domain of WT, **+1 fs** and **–1 fs** IbsC; (**J**) changes of membrane integrity upon WT, **+1 fs** and **–1 fs** IbsC expression. Control cells (with empty vector) and cells expressing WT, **+1 fs** and **–1 fs** IbsC were analyzed by flow cytometry following stained with DiBAC4(3). The percentage of cells with a depolarized membrane was evaluated in the region denoted M1. All these assays were repeated at least three times independently and data shown is representative one of three independent experiments.

IbsC was first identified in *E. coli* K-12 as part of the ibsC/sibC toxin–antitoxin system. It encodes a highly hydrophobic 19-amino-acid protein which could be anchored in the inner membrane of *E. coli*, and its overexpression is deleterious to *E. coli* by compromising membrane's integrity and resulting in membrane depolarization (3,12,13). Extensive mutations can be tolerated by *ibsC* gene without loss of toxicity, and the fusion of other proteins at its N terminus could be tolerable without toxicity compromising (14). In our previous work of the intracellular selection of *trans*-cleaving hammerhead ribozymes by using *ibsC* as a reporter gene in *E.coli* (15), we found that expression of **–1 fs** of *ibsC* would cause the death of *E. coli* unexpectedly. The **–1 fs** was constructed by deleting the first two nucleotides of the coding sequence of *ibsC* (Figure 2A) and fusion-expressed with EGFP (vector **2** with ampicillin resistance, Figure 2B). As shown in Figure 2C, the IPTG-induced expression of the fusion protein (EGFP/–1 fs) completely inhibited the growth of *E. coli* cells on the agar plate, which is consistent with the result of wild-type (WT) *ibsC* (vector **1**, Figure 2B). To confirm these observations, the **–1 fs** mutant of *ibsC* was independently constructed into downstream of T7 promoter and lacO operon on a new expression plasmid (vector **3**, Figure 2B). Again, the **–1 fs** mutant inhibited the cellular growth effectively as WT *ibsC*, and the growth curves in liquid LB were consistent with the results of agar plate (Supplementary Figure S1). However, because of the use of restriction endonuclease site of *Bam*HI for inserting the target gene, there are two extra amino acid residues in the N-terminal of **–1 fs** protein, and meanwhile the original stop codon (TAA) in original *ibsC* gene cannot terminate the translation of **–1 fs** protein due to the frameshift mutation. Therefore, we re-constructed the plasmid vector by removing the *Bam*HI site and inserting a TAA stop codon adjacent to the original position (vector **4**, Figure 2B). The overexpression of **–1 fs** with stop codon did inhibit the growth of *E. coli* as expected (Figure 2E). As the frameshift mutation occurred at 5′ end of the coding sequence, the protein sequence of **–1 fs** mutant changed completely compared with that of WT IbsC, while the mRNA sequence of **–1 fs** mutant has almost no change except for two missing nucleotides (Supplementary Figure S2). To verify that the toxicity is caused by **–1 fs** protein rather than RNA, plasmid vectors with inserted genes (**–1 fs** or WT of *ibsC*) that can only be transcribed but not be translated due to the deletion of RBS and start codon ATG, were constructed (vector **5**, Figure 2B). Cells transformed by the vectors grew much well on the culture plate without or with IPTG induction (Figure 2F and G), thus excluding the possibility of the toxicity caused by RNA. Unlike WT protein of *ibsC* with functional domain near C-terminal and middle of the protein sequence, we found that all amino acid residues of **–1 fs** protein are essential for its toxicity after systematical deletion analysis of two proteins sequence respectively (Supplementary Figures S3 and S5) (14).

As the **–1 fs** mutant of ibsC retains toxicity, we were very curious whether the **+1 fs** protein is a toxin too. Thus, the **+1 fs** gene was constructed by deleting the first nucleotide of the coding sequence of *ibsC* (Figure 2A), which was then inserted into the plasmid vector (Figure 2H). The expression of **+1 fs** protein induced cell death like **–1 fs** and WT proteins as well (Supplementary Figure S1). It was worth noting that a TAA stop codon close to 3′-terminal of **+1 fs** gene was yielded due to the frameshift mutation (Figure 2A). Therefore, we deleted the extra sequence after this TAA stop codon and found that this mutant still retained its toxicity to inhibit the cellular growth on the agar plate (Supplementary Figure S4). Although this **+1 fs** mutant could be a new toxin, two opal stop codons (TGA at first and eighth site) appeared in its coding sequence due to the frame-shifted mutation (Figure 2H). As previous studies has shown that TGA was the leakiest stop codon (16), we speculated that the **+1 fs** mutant could be fully expressed through ribosomal readthrough (17) and the full-length **+1 fs** proteins caused the death of E. coli. Therefore, the two internal stop codons of **+1 fs** were changed into the codes of common 20 amino acids systematically to investigate the potential inserted amino acid residues (AAR) during ribosomal readthrough. As shown in Figure 2H, most of all the 400 variants of **+1 fs** mutant revealed toxicity to inhibit the colony formation on the agar plate, in which around 69% of mutants containing at least 50% toxicity to inhibit cell growth. These results explained that overexpressed **+1 fs** gene with two stop codons still caused the death of *E. coli*. In addition, a mutant **+1 fs** (SS) with 100% toxicity was picked out for the deletion analysis of protein sequence. We found that the N-terminal and midterm of **+1 fs** protein was important for its toxicity, which was different from **–1 fs** or WT protein of *ibsC* (Supplementary Figures S4 and S6) (14).

Though all the proteins of **–1 fs**, **+1 fs** and WT proteins of IbsC gene have been proved to be toxic to *E. coli* cells, we hardly believe that all three different proteins work with the same mechanism of type I toxins, namely through disrupting inner membrane integrity of bacteria, because the 5′ frameshift mutation would led to tremendous changes in protein properties. As shown in Figure 2I, the three proteins differed not only in length and molecule weight (MW), but also in the physicochemical properties such as net charge, isoelectric point (p*I*) and hydrophobicity. Traditionally, the accumulation of error proteins due to frameshift mutations may also cause intracellular energy waste to affect the cellular normal growth. In addition, the acute changes of protein properties caused by the frameshift mutation, such as PI and hydrophobicity, may lead to cytotoxicity to inhibit the cellular growth (11). Therefore, a commonly used fluorescent dye DiBAC4(3) for the detection of membrane integrity was applied to stain the *E. coli* cells which had been transformed to express the three different proteins respectively. As an indicator of membrane depolarization, DiBAC4(3) had been used to evaluate the detrimental effects of Type I toxins on the membrane (3), because this dye can only permeate through depolarized cell membranes and binds to intracellular cytoplasmic proteins to emit enhanced fluorescence (18). After induced expression for 3 h by adding IPTG, *E. coli* cells with different vectors were incubated with DiBAC4(3) for 20 min and then analyzed by flow cytometry. As shown in Figure 2J, no changes were observed for the control with empty vector. In contrast, the induced expression of WT, **–1 fs** and **+1 fs** proteins of *ibsC* had rapid and dramatic effects of membrane depolarization, yielding 80.1%, 89.7% and 63.2% of fluorescent cells respectively (Figure 2J). This implied that **–1 fs** and **+1 fs** proteins of *ibsC* have the same function as WT protein to arrest the cellular growth by impairing the membrane's integrity.

### Toxicity of frameshift mutations of other toxin genes

To determine whether our unexpected findings of *ibsC* was an individual event or ubiquitous phenomenon, several other toxins including *dinQ*, *tisB*, *ldrD*, *pndA*, *flmA* and *ghoT*, were selected to test the toxicity of their frameshift mutations. *dinQ*, *tisB*, *ldrD*, *pndA* and *flmA* are all Type I TA toxins and encode 27, 29, 35, 50 and 52 amino acids respectively. Overexpression of these toxins will depolarize the cell membrane and decrease the intracellular ATP concentration to suppress cell growth (5,6,19–22). *ghoT* was first identified type V toxin-antitoxin system in which a protein antitoxin inhibits the toxin by cleaving its mRNA specifically; *ghoT* codes a membrane lytic peptide, 57 AAR in length, which could cause damage to the cell membranes and induce a persistence (23).

**Figure 3.**
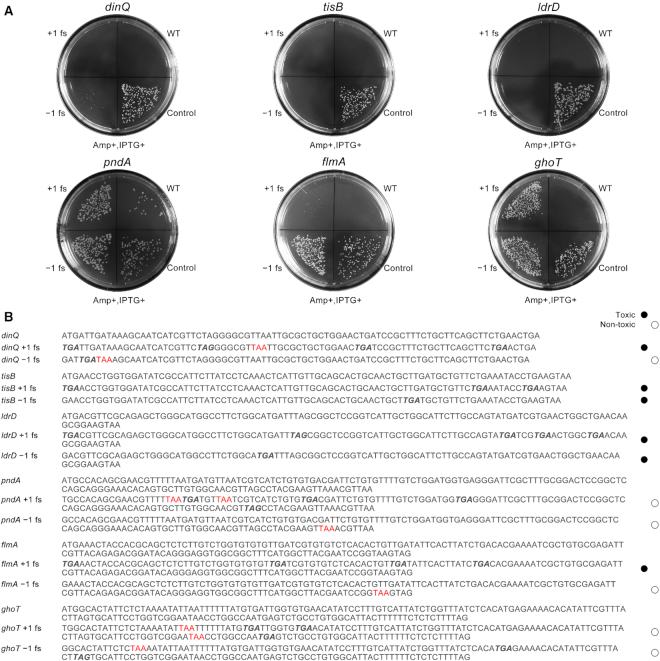
Toxicity of frameshift mutations of other toxins: (**A**) toxicities of fusion expressed WT toxin gene and its frameshift mutants on agar plate; (**B**) DNA sequence alignment of WT toxin gene and its frameshift mutants. TAA stop codon is highlighted in red, TAG and TGA are bold italics.

All the toxin genes and its two types of frameshift mutations were constructed into expression vectors and their toxicity was tested on agar plate as well as in liquid culture medium. As shown in Figure 3A, surprisingly, both **+1 fs** and **–1 fs** of TisB and LdrD kept the toxicity to significantly inhibit cell growth. The **+1 fs** mutants of DinQ and FlmA suppressed the growth of *E.coli* like WT protein, but their **–1 fs** mutants showed decreased toxicity relatively. And, we also found that both **+1 fs** and **–1 fs** mutants of PndA and GhoT lost its toxicity significantly. The results of growth curve lines were consistent with the observations on agar plates (Supplementary Figures S7–S12). As shown in Figure 3B, in all 12 frameshift mutants of toxins, six mutants (**+1 fs** and **–1 fs** of TisB and LdrD, **+1 fs** of DinQ and FlmA) retained toxicity, while other six mutants (**–1 fs** variant of DinQ and FlmA, **+1 fs** and **–1 fs** variant of PndA and GhoT) lost toxicity. After analyzing the coding sequence of these genes, we found that at least one stop codon TAA was yielded in all the six mutants without toxicity, while five mutants with toxicity contain no TAA stop codon (Figure 3B). As the order of termination efficiency of the stop codons is TAA>TAG>TGA (17), the TAA stop codon generated by the frameshift mutation can terminate the translation most effectively, thus it is reasonable that six mutants with internal TAA lost its toxicity. The only exception, –1 fs of DinQ1 (Figure 3B), was found that its functional domain was near N-terminal of protein sequence, which explains why its toxicity had not been interrupted by the newly generated TAA stop codon (Supplementary Figure S13). Therefore, based on these results we speculated that the lack of stop codon TAA in full-length open reading frame (ORF) may be a predictor for us to find more genes whose frameshift mutants could retain function.

### Function of the frame-shifted DfrB3 proteins

**Figure 4.**
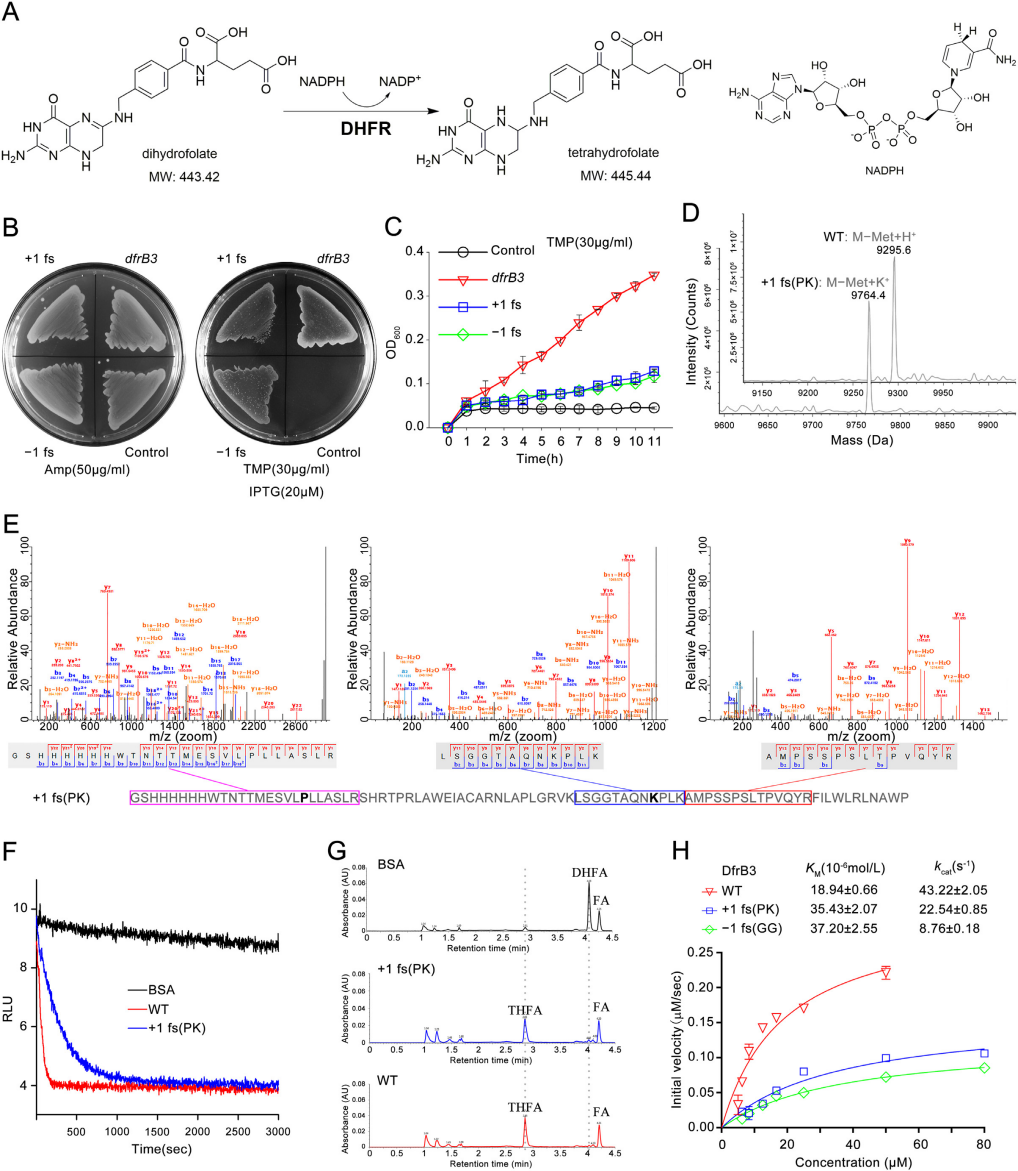
Frameshift mutation of *dfrB3* gene: (**A**) the DHFR-catalyzed reduction of dihydrofolate to tetrahydrofolate by using NADPH; (**B**) resistance of WT, **+1 fs** and **–1 fs** mutant of *dfrB3* to antibiotic TMP on agar plate; (**C**) growth curve lines of *E.coli* containing WT, **+1 fs** and **–1 fs** mutant of *dfrB3* in the presence of TMP in liquid LB medium; (**D**) ESI-TOF MS analysis of WT and **+1 fs** (PK) proteins of *dfrB3*; (**E**) tandem MS analysis of the trypsin-digested fragments of **+1 fs** (PK) protein, indicating their sequences are consistent with the expectation; (**F**) fluorescent tracking of the reduction reactions catalyzed by BSA as well as WT and **+1 fs** (PK) proteins of *dfrB3* at 460 nm (for more detail see Supplementary Figure S25); (**G**) UPLC–MS analysis of the reduction reactions catalyzed by BSA as well as WT and **+1 fs** (PK) proteins of *dfrB3*, and extracted ion chromatograms and mass spectra are shown in Supplementary Figure S21–23. FA = folate, DHFA = dihydrofolate, THFA = tetrahydrofolate; H, kinetic characterization of WT, **+1 fs** (PK) and **–1 fs** (GG) proteins of *dfrB3*. Solid lines show best fit to the Michaelis–Menten equation. All these assays were repeated at least three times independently and data shown is representative one of three independent experiments.

To verify our hypothesis, we searched the Genebank from NCBI to find genes which lack stop codon TAA in coding sequence. Then, a larger antibiotic resistance gene *dfrB3* was identified, and it is an integron-encoded dihydrofolate reductase (DHFR) found in *Klebsiella oxytoca* (24). DHFR catalyzes the reduction of dihydrofolate to tetrahydrofolate using NADPH as reductant (Figure 4A), and tetrahydrofolate is a critical co-enzyme for the synthesis of purines, thymidylic acid, and certain amino acids (25). Trimethoprim (TMP) is an antibiotic that can bind to the active site of the chromosomal DHFR to inhibit the synthesis of tetrahydrofolate in bacteria. However, the expression of exogenous *dfrB3* gene will induce antibiotic resistance because the TMP cannot inhibit the *dfrB3*-encoded DHFR enzyme. In the ORF of *dfrB3* gene, we could not find any internal TAA code in neither +1 nor –1 reading frame. Therefore, WT, **+1 fs** and **–1 fs** sequence of *dfrB3* were constructed into expression vectors and induced to express in *E. coli* on agar plate as well as in liquid culture medium with 30 μg/ml TMP. As shown in Figure 4B and C, all three proteins allowed *E. coli* to survive in the presence of antibiotics, while *E. coli* transfected with empty vector was unable to grow on the same agar plate. This result suggested that **+1 fs** and **–1 fs** proteins of *dfrB3* could catalyze the reduction of dihydrofolate like the WT enzyme to provide the indispensable tetrahydrofolate for the cellular growth of bacteria. Therefore, we decided to use the pure **+1 fs** and **–1 fs** DfrB3 proteins to verify their catalytic activity. But both frameshift genes contain two weak stop codons respectively: **+1 fs** has a TAG (11th) and a TGA (50th), while **–1 fs** has two TGA (52th and 74th). To obtain the pure frame-shifted proteins, we changed the stop codons (TAG and TGA) into the code of one common amino acid in turn, and finally screened out the active mutants without internal stop codon. When the TAG and TGA of **+1 fs** gene were modified to CCT (P) and AAA (K) respectively, the mutant **+1 fs** (PK) protein still retained the TMP resistance (Supplementary Figure S14). A poly-histidine tag was engineered at the amino-terminus of **+1 fs** (PK) protein without compromising its antibiotic activity, and then the **+1 fs** (PK) protein was purified by affinity chromatography (Supplementary Figure S15). The purified **+1 fs** (PK) protein was analyzed by electrospray ionization time-of-flight MS (ESI-TOF MS), yielding a peak observed at 9764.4 Da corresponding to the frame-shifted protein lacking the initiating Met (M-Met+K^+^: expected 9758.4 Da, Figure 4D and Supplementary Figure S17). When the WT DfrB3 was analyzed under the identical condition, a peak at 9295.6 Da of wild-type protein was obtained (M-Met+H^+^: expected 9296.3 Da, Figure 4D and Supplementary Figure S16). Furthermore, **+1 fs** (PK) protein was digested with trypsin and analyzed by tandem MS (Figure 4E and Supplementary Figure S19), a series of y and b ions of three digested peptides fragments clearly revealed that their sequences are consistent with the expectation, verifying that **+1 fs** (PK) protein is the frame-shifted DfrB3 chemically.

The WT DfrB3 utilizes NADPH as the reductant to reduce dihydrofolate, yielding NADP as the by-product. Because NADPH differs from NADP in fluorescence properties, the *in vitro* reduction reactions containing dihydrofolate and NADPH could be traced by measuring the fluorescent intensity at 460 nm. As illustrated in Figure 4F, the control protein (BSA) could not cause the obvious fluorescent change of reaction mixture containing dihydrofolate and NADPH, whereas both WT and **+1 fs** (PK) proteins of *dfrB3* caused the sharp decrease of fluorescence of the reaction system in 10 min, indicating that the NADPH was consumed rapidly. To some extent, this result proved that the frame-shifted protein of **+1 fs** (PK) could have a similar catalytic ability to the DHFR enzyme. Therefore, in order to obtain the direct evidence, UPLC–MS was used to analyze the reduction reaction of dihydrofolate with NADPH catalyzed by the **+1 fs** (PK) protein. As shown in Figure 4G and Supplementary Figure S23, the signals were assigned to the respective entities by ESI-MS analysis, and much to our delight we observed the disappearance of the signal of dihydrofolate, as well as the appearance of a signal of tetrahydrofolate in the reaction mixture, which is consistent with the catalytic result of WT DfrB3 protein (Figure 4G and Supplementary Figure S22). Meanwhile, no signal of tetrahydrofolate was detected when BSA protein was used in the same reaction (Figure 4G and Supplementary Figure S21). All those results clearly indicate that the **+1 fs** (PK) protein can catalyze the reduction of dihydrofolate to tetrahydrofolate like the dihydrofolate reductase encoded by WT *dfrB3* gene

Subsequently, a **–1 fs** mutant of *dfrB3*, called **–1 fs** (GG) without stop codons in the ORF, was selected out, and its encoded protein was purified and then identified with ESI-TOF and tandem MS (Supplementary Figures S14, S18 and S20). The purified **–1 fs** (GG) revealed the same catalytic ability to reduce dihydrofolate with NADPH, which was also confirmed by UPLC–MS analysis (Supplementary Figure S24), showing both +1 and –1 frameshift mutants of *dfrB3* gene encodes proteins that have the same function as WT enzyme. However, the catalytic efficiency of three enzymes is apparently different from each other according to the fluorescence-tracing results (Supplementary Figure S25). In order to quantify these observations, we determined the efficiencies (*k*_cat_*= v*_max_/*K*_M,_*v*_max_ = the maximum rate of the enzyme reaction, *K*_M_ = the Michaelis constant) of the reduction reactions catalyzed by WT, **+1 fs** (PK) and **–1 fs** (GG) DfrB3 proteins (Figure 4H and Supplementary Figure S26). The quantitative analyses revealed **+1 fs** (PK) and **–1 fs** (GG) have about 1/2 and 1/5 of the catalytic efficiency of WT enzyme respectively (Figure 4H). This result might explain why the WT *dfrB3* gene is preserved in bacteria, while the other two frameshift genes are hidden.

## DISCUSSION

A textbook gene encodes a protein using a single reading frame. Since the DNA language has three-nucleotide ‘letters’ per codon ‘word’ and DNA consists of two strands, up to six reading frames are possible at a given gene locus. Overlapping genes have been known since the beginning of virus complete genome sequencing, as exemplified by the gene B of the single-stranded DNA bacteriophage φX174, which is completely contained within gene A (26). In 1984, Ohno discovered a protein in Flavobacteria with the capacity to degrade nylon, and this protein is coded within a previously existing gene, but in an alternative reading frame (27). Although an experimental verification of two protein-coding genes in the same DNA locus is extremely challenging, an increasing number of nontrivially overlapping genes in prokaryotes have been found over the last few decades (28). However, the genes described here were obviously different with the known overlapping genes: (i) the proteins encoded by alternative reading frames (+1 or –1) have the same function as the wild-type one, while all the known overlapping genes encoded proteins with different functions; (ii) the function of frame-shifted proteins is hidden because of the lack of the corresponding efficient start codons, while the overlapping genes usually have their own start and stop codons.

The frameshift mutation is generally considered to be lethal because it could result in radical changes of the chemical composition of the protein sequence behind. On one hand, the unexpected results mentioned above is totally out of our imagination according to the existing knowledge, but on the other hand, from an evolutionary point of view, the observations might seem reasonable. As a central pillar of the modern evolutionary theory, all organisms now living on the earth are believed to have a last universal common ancestry (LUCA), which was probably highly error prone to evolve into different species. Thus, if the proteins encoded by a given gene from different reading frames have the same function, LUCA could effectively resist the instability caused by the frameshift mutations in reproduction, especially those genes that are crucial to the survival of LUCA and its descendants. As described in our research, both TA systems and the synthesis of tetrahydrofolate are essential for the survival of bacteria under stressed conditions. As organisms continue to become more refined and precise during evolution, the protein with the best catalytic efficiency could finally win out, and other protein-coding sequences from different reading frames would be hidden consequently. A possible way to find out those hidden genes is proposed by using the absence of extra TAA in ORF as a clue, because internal TAA means that the frame-shifted protein has no function at all, or their function is no longer needed in any case. Based on this hypothesis, we indeed found new hidden genes, but it would face enormous challenges because the percentages of genes without internal TAA varies greatly in different bacteria, such as 1.3% in *E. coli* O157 versus 71.8% in *Paeruginosa* PAO1. Furthermore, although certain frame-shifted proteins in prokaryotes were verified to retain the same function as the WT protein here, whether the described phenomenon exists in eukaryotes needs to be questioned and explored.

## Supplementary Material

gkaa169_Supplemental_File
