# Supplementary material for: Frame-shifted proteins of a given gene retain the same function
Source: Nucleic Acids Res. 2020 Mar 18;48(8):4396–404. doi: 10.1093/nar/gkaa169 (PMC7192591; doi:10.1093/nar/gkaa169)
Supplement: gkaa169_Supplemental_File [file gkaa169_supplemental_file.pdf]

Supplementary Information

Frame-shifted proteins of a given gene retain the same function

Xin Huang,[1] Rong Chen,[2] Meiling Sun,[1] Yan Peng,[1] Qinlin Pu,[1] Yi Yuan,[1] Gangyi Chen,[1] Juan Dong,[1] Feng Du,[1] Xin Cui,[1] and Zhuo Tang[1]*

[1]Natural Products Research Center, Chengdu Institution of Biology, Chinese Academy of Science, Chengdu 610041, P. R. China.

[2]Ethnomedicine College, Chengdu University of Traditional Chinese Medicine, Chengdu 610041, P. R. China.

*Correspondence author. Email: tangzhuo@cib.ac.cn

Table of Contents

Supplementary Information 1

Frame-shifted proteins of a given gene retain the same function 1

1. Supplementary text 3

1.1. IbsC 3

1.1.1. Toxicity of frame-shifted protein and RNA of *ibsC* 3

1.1.2. RNA and protein sequences of frame-shifted proteins and *ibsC* 4

1.1.3. Confirmation of minimized sequence of −1 fs 4

1.1.4. Confirmation of minimized sequence of +1 fs 5

1.1.5. Contribution of single amino acids at given position to toxicity of −1 fs 5

1.1.6. Contribution of single amino acids at given position to toxicity of +1 fs 6

1.2. Other toxin genes 7

1.2.1. Toxicity of frameshift mutations of other toxin genes 7

1.2.2. Influence of substitution of TGA by TAA on toxicity of *dinQ* +1 fs 9

1.3. DfrB3 10

1.3.1. Stop codon substitution in +1 fs and −1 fs mutant of dfrB3 10

1.3.2. Purification and PAGE analysis of frame-shifted protein 10

1.3.3. Molecular weight determination by ESI-TOF MS 11

1.3.4. Analysis of AAR compositions of protein by LC-MS/MS 12

1.3.5. Catalytic products analysis by UPLC-MS 14

1.3.6. Fluorescent tracking of the reduction reactions catalyzed by DfrB3, +1 fs (PK) and −1 fs(GG) ... 17

1.3.7. Kinetic characterization of DfrB3, +1 fs (PK) and -1 fs (GG) 17

2. Gene and protein sequences used in this study 18

2.1. Gene sequences 18

2.2. Protein sequences 19

3. Supplementary Table 20

3.1. Supplementary Table S1. Primers of plasmid construction 20

3.2. Supplementary Table S2. Protein sequences of WT proteins and its frameshift mutations 21

4. References 23

1. Supplementary text

1.1. IbsC

1.1.1. Toxicity of frame-shifted protein and RNA of *ibsC*

The −1 fs mutant of *ibsC* was independently constructed into downstream of T7 promoter and lacO operon on a new expression plasmid. −1 fs mutant inhibited the cellular growth effectively as WT IbsC on agar plate and liquid LB medium (Figure S1).

Now that the −1 fs mutant of *ibsC* retains toxicity, we were very curious if the +1 fs protein is a toxin too. Thus, the +1 fs gene was constructed by deletion of the first nucleotide of coding sequence of *ibsC* and inserted into the plasmid vector. The expression of +1 fs protein also caused the dead of *E.coli* like that of −1 fs and WT IbsC on agar plate and liquid LB medium (Figure S1).

Due to the frameshift mutation occurred at 5' end of coding sequence, the protein sequence of +1 fs and −1 fs mutant changes completely, while the mRNA sequence of frameshift mutant has almost no change compared with that of WT *ibsC* except for first one or two missing nucleotides (Figure S2). To verify that the toxicity of frameshift mutations is caused by protein rather than RNA, we constructed recombinant plasmid vectors with inserted genes to transcribe RNA only due to the deletion of translation elements including RBS and start codon ATG (Figure S1). Cells transformed by the vectors grew much well on the culture plate without or with IPTG induction, and the growth curve in liquid LB were consistent with results of agar plate (Figure S1).

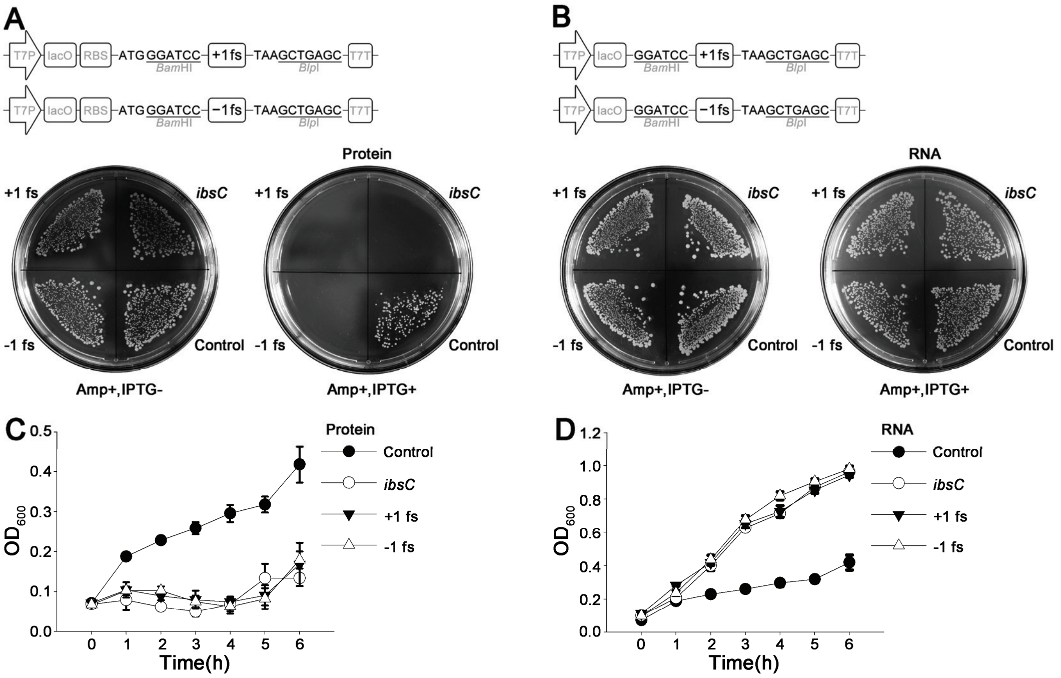

Figure S1. Toxicity of frame-shifted protein and RNA of *ibsC*: A and B, the toxicity of *ibsC* and frameshift mutants were evaluated on LB agar plate without and with IPTG; C, the proteins toxicity of *ibsC* and frameshift mutants were assessed in liquid medium; D, the RNA toxicity of *ibsC* and frameshift mutants were tested in liquid medium. The experiments were performed at least in triplicate and data shown is representative one of three independent experiments.

1.1.2. RNA and protein sequences of frame-shifted proteins and *ibsC*

Due to deletion of fist one or two nucleotide at 5' end of coding sequence, mRNA sequence of frameshift mutant has almost no change except for first one or two missing nucleotides, while the protein sequence of -1 fs and +1 fs mutant changes completely compared with that of WT IbsC (Figure S2). However, the overexpression of +1 fs and −1 fs still led to growth inhibition on solid medium plate. Therefore, it is very interesting to validate the functional sequence of +1 fs and −1 fs.

```
IbsC   AUG AUG CGA CUU GUC AUC AUA CUG AUU GUA CUG UUA CUC AUA AGU UUC AGC GCU UAU UAA
        M   M   R   L   V   I   I   L   I   V   L   L   L   I   S   F   S   A   Y   -
-1 fs    GAU GCG ACU UGU CAU CAU ACU GAU UGU ACU GUU ACU CAU AAG UUU CAG CGC UUA UUA A...
          D   A   T   C   H   H   T   D   C   T   V   T   H   K   F   Q   R   L   L
+1 fs   UGA UGC GAC UUG UCA UCA UAC UGA UUG UAC UGU UAC UCA UAA GUU UCA GCG CUU AUU AA...
         -   C   D   L   S   S   Y   -   L   Y   C   Y   S   -   V   S   A   L   I
```

Figure S2. RNA and protein's sequence alignment of *ibsC*, −1 and +1 mutants: TAA stop codon is highlighted in red, TGA stop codon is labeled in blue, and ATG start codon is labeled in green.

1.1.3. Confirmation of minimized sequence of −1 fs

It is not clear that all the AARs are necessary to the toxicity of −1 fs. A serial of truncation mutants of −1 fs were constructed by removing N- or C-terminal of ORF. BL21(DE3) competent cell were transformed by these mutants and recovered 1 h in LB medium, following equal bacteria solution inoculated on culture plate containing IPTG for overnight. Culture without IPTG induction was used as control to monitor the viability of transformed cells.

As showed in Figure S3, removal of last 1 or 2 AARs near C-terminal of −1 fs caused cell proliferation on culture plate induced by IPTG. The observations declared that AARs in the C-terminal is integrant to its toxicity. Therefore, no more AARs adjacent to C-terminal of −1 fs were deleted to confirm the toxic core of −1 fs. Then 1 to 5 codons in the 5' end of −1 fs were removed. *E.coli* including these mutant formed clear bacterial colonies on the culture plate, which indicated that the AARs in the N-terminal −1 fs is also key functional fragments for maintaining of toxicity.

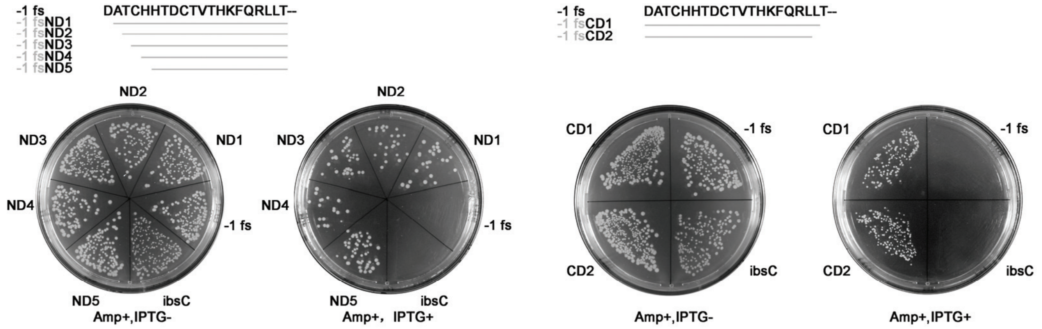

Figure S3. Characterization of N-terminal and C-terminal of −1 fs: protein's sequence alignment of truncated -1 mutants and toxicities of −1 fs mutants were assessed on LB agar plate without and with IPTG.

1.1.4. Confirmation of minimized sequence of +1 fs

To confirm the functional domain of +1 fs, a serial of truncation mutants of +1 fs were constructed by removing N- or C-terminal of ORF. 1 to 5 codons in the 5' or 3' end of +1 fs were deleted respectively. BL21(DE3) competent cell were transformed by these mutants and recovered 1 h in LB medium, following equal bacteria solution inoculated on culture plate containing IPTG for overnight. Culture without IPTG induction was used as control to monitor the viability of transformed cells.

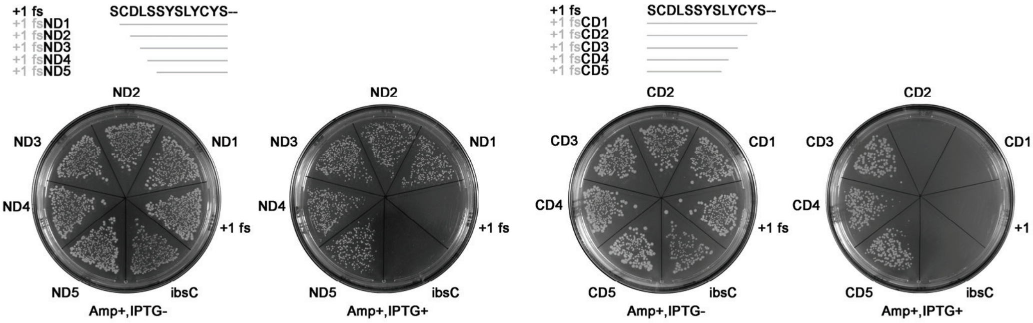

Figure S4. Characterization of N-terminal and C-terminal of +1 fs: protein's sequence alignment of truncated +1 mutants and toxicities of +1 fs mutants were assessed on LB agar plate without and with IPTG.

As showed in Figure S4, loss of 1 and 2 AARs in the C-terminal of +1 fs suppress the growth of *E.coli*, implying that the two AARs close to C-terminal of +1 fs is not significant to its toxicity. But, colony formations on culture plate were observed when 3 to 5 AARs in the C-terminal of +1 fs were depleted. Furthermore, deletion of 1 to 5 AARs in N-terminal of +1 fs caused colony formations as observed with elimination of 3 to 5 AARs in the C-terminal of +1 fs. The results suggested that AARs in the N-terminal and midterm of +1 fs is important for its toxicity. The property of +1 fs is different from that of WT IbsC with functional domain near C-terminal and middle of sequence (1).

1.1.5. Contribution of single amino acids at given position to toxicity of −1 fs

The whole 20 AARs of −1 fs is necessary to its toxicity. However, the role of each amino acid at given position on toxicity of −1 fs is not clear. 20 deletion mutants of −1 fs were constructed. The variations were transformed into BL21(DE3) cells following equal bacteria solution inoculated on culture plate to assess its toxicity.

As shown in Figure S5, last single AAR deletion of −1 fs abolished its toxicity to a large extent. However, the elimination of single AAR at 1st, 2nd, 14th, 16th and 17th site could not suppress the growth of transformed *E.coli* completely.

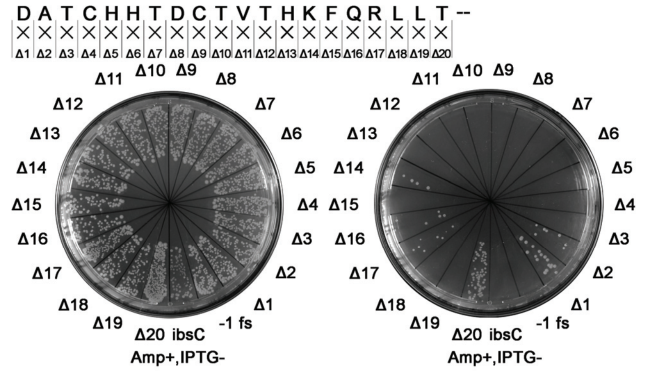

Figure S5. Influence of single AAR deletion on the toxicity of frame-shifted protein: toxicities of −1 fs mutants were assessed on LB agar plate without and with IPTG.

1.1.6. Contribution of single amino acids at given position to toxicity of +1 fs

The functional core sequence focus on AARs from position 1 to 13 of +1 fs. To evaluate the role of each amino acid at given position on toxicity of +1 fs, 13 deletion mutants of +1 fs were constructed respectively. The variations were transformed into BL21(DE3) cells following equal bacteria solution inoculated on culture plate to assess its toxicity.

As shown in Figure S6, single deletion of 1st or 2nd AAR near N-terminal of +1 fs led to normal proliferation of transformed bacterium even in the induction of IPTG. In the induction of IPTG, the removal of single AAR ranged from 3 to 13 restrain bacterial growth when mutants were induced to overexpress. The observations implied that the first two AARs is very important to +1 fs's toxicity. +1 fs could tolerate extensive AAR deletion except the first two AARs close to N-terminal.

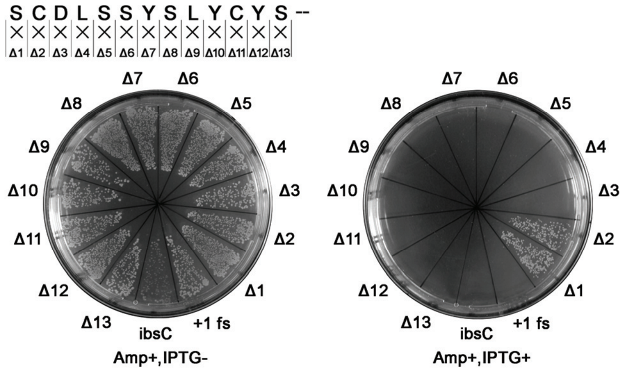

Figure S6. Influence of single AAR deletion on the toxicity of frame-shifted protein: toxicities of +1 fs mutants were assessed on LB agar plate without and with IPTG.

1.2. Other toxin genes

1.2.1. Toxicity of frameshift mutations of other toxin genes

The genes and its frameshift mutations of dinQ, tisB, ldrD, pndA, flmA and ghoT were constructed into expression vector and the toxicity were tested on agar plate and liquid culture medium.

The +1 fs mutations of *dinQ* suppressed the growth of *E.coli* like WT protein, but −1 fs mutations of *dinQ* showed decreased toxicity compared with WT protein (Figure S7). The results of growth curve lines were consistent with the observations on agar plates.

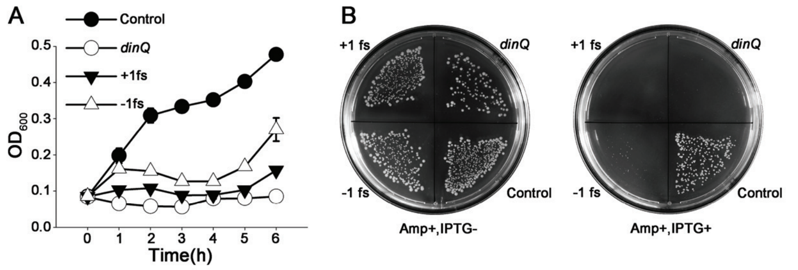

Figure S7. Toxicity of frameshift mutations of *dinQ*: A, the proteins toxicity of *dinQ* and frameshift mutants were assessed in liquid medium; B, the toxicity of *dinQ* and frameshift mutants were evaluated on LB agar plate without and with IPTG. The experiments were performed at least in triplicate and data shown is representative one of three independent experiments.

The +1 fs and -1 mutations of *tisB* suppressed the growth of *E.coli* like WT protein (Figure S8). The results of growth curve lines were consistent with the observations on agar plates.

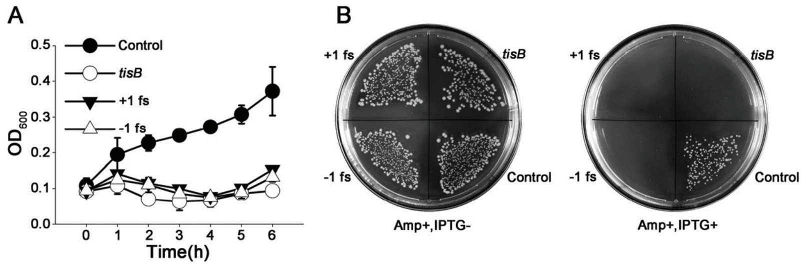

Figure S8. Toxicity of frameshift mutations of *tisB*: A, the proteins toxicity of *tisB* and frameshift mutants were assessed in liquid medium; B, the toxicity of *tisB* and frameshift mutants were evaluated on LB agar plate without and with IPTG. The experiments were performed at least in triplicate and data shown is representative one of three independent experiments.

The +1 fs and -1 mutations of *ldrD* suppressed the growth of *E.coli* like WT protein (Figure S9). The results of growth curve lines were consistent with the observations on agar plates.

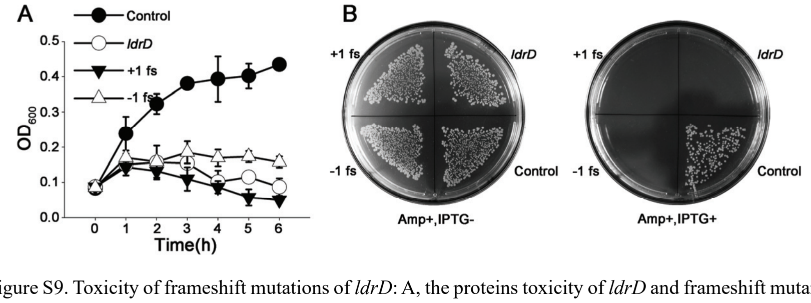

Figure S9. Toxicity of frameshift mutations of *ldrD*: A, the proteins toxicity of *ldrD* and frameshift mutants were assessed in liquid medium; B, the toxicity of *ldrD* and frameshift mutants were evaluated on LB agar plate without and with IPTG. The experiments were performed at least in triplicate and data shown is representative one of three independent experiments.

The +1 fs and −1 fs mutant of *pndA* loss its toxicity significantly (Figure S10). The results of growth curve lines were consistent with the observations on agar plates.

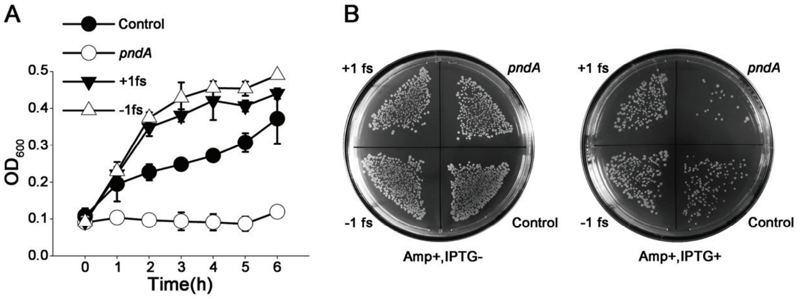

Figure S10. Toxicity of frameshift mutations of *pndA*: A, the proteins toxicity of *pndA* and frameshift mutants were assessed in liquid medium; B, the toxicity of *pndA* and frameshift mutants were evaluated on LB agar plate without and with IPTG. The experiments were performed at least in triplicate and data shown is representative one of three independent experiments.

The +1 fs mutations of *flmA* suppressed the growth of *E.coli* like WT protein, but −1 fs mutations of *flmA* showed decreased toxicity compared with WT protein (Figure S11). The results of growth curve lines were consistent with the observations on agar plates.

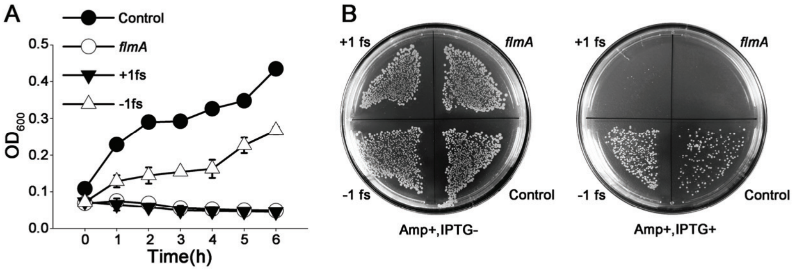

Figure S11. Toxicity of frameshift mutations of *flmA*: A, the proteins toxicity of *flmA* and frameshift mutants were assessed in liquid medium; B, the toxicity of *flmA* and frameshift mutants were evaluated on LB agar plate without and with IPTG. The experiments were performed at least in triplicate and data shown is representative one of three independent experiments.

The +1 fs and −1 fs mutant of *ghoT* loss its toxicity significantly (Figure S12). The results of growth curve lines were consistent with the observations on agar plates.

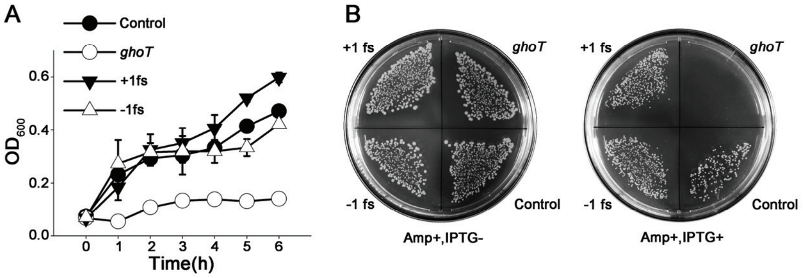

Figure S12. Toxicity of frameshift mutations of *ghoT*: A, the proteins toxicity of *ghoT* and frameshift mutants were assessed in liquid medium; B, the toxicity of *ghoT* and frameshift mutants were evaluated on LB agar plate without and with IPTG. The experiments were performed at least in triplicate and data shown is representative one of three independent experiments.

1.2.2. Influence of substitution of TGA by TAA on toxicity of *dinQ* +1 fs

dinQ +1 fs owned a TAA stop codon in the middle of the coding sequence. However, *dinQ* +1 fs still maintained toxicity to inhibit the growth of *E.coli* cells. To confirm whether the function domain of *dinQ* +1 fs is near the N-terminal, we truncated the *dinQ* +1 fs in the TAA stop codon caused by frameshift mutation. We found that the truncated +1 fs (termed as +1 fs S) indeed maintained its toxicity as +1 fs and WT protein. (Figure S13).

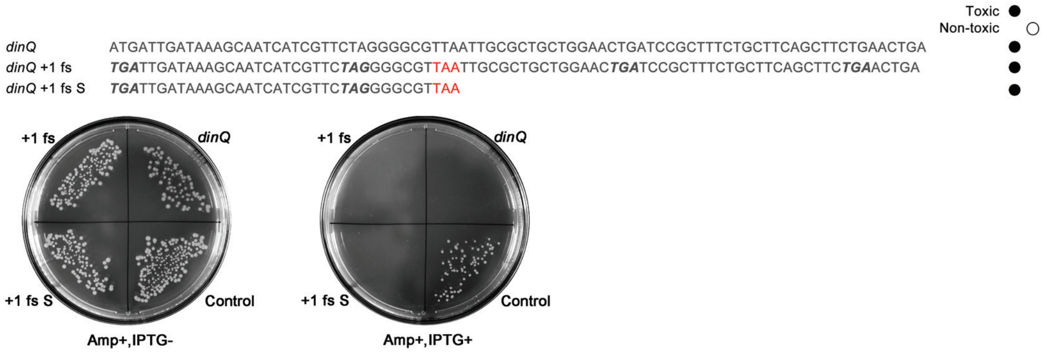

Figure S13. Influence of substitution of TGA by TAA on toxicity of *dinQ* +1 fs. The toxicity of DinQ +1 fs S was evaluated on LB agar plate without and with IPTG. The experiments were performed at least in triplicate and data shown is representative one of three independent experiments.

1.3. DfrB3

1.3.1. Stop codon substitution in +1 fs and −1 fs mutant of dfrB3

To obtain the pure frame-shifted proteins, we changed the stop codons (TGA and TAG) into the code of one common amino acid in turn, and finally screened out the active mutants without internal stop codon. We found that +1 fs(PK) and −1 fs(GG) showed resistant to TMP as +1 fs and −1 fs, respectively (Figure S14).

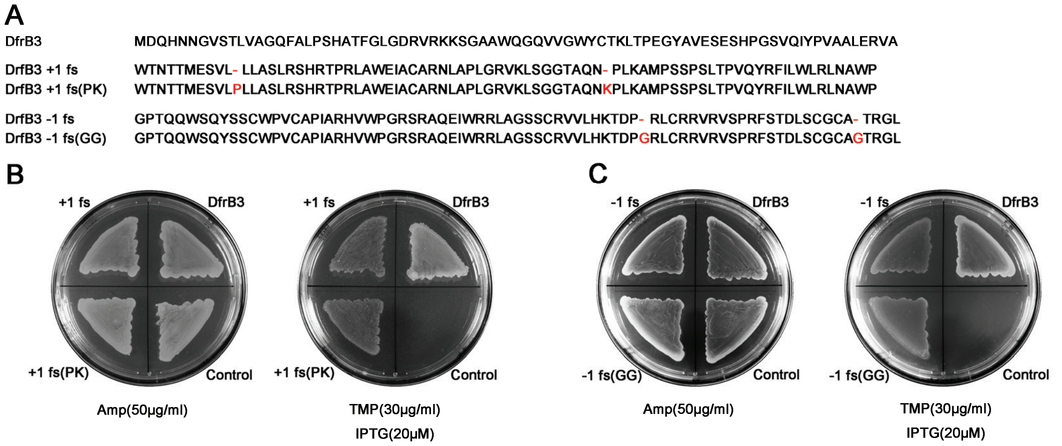

Figure S14. Stop codon substitution in +1 fs and −1 fs mutant of dfrB3: A, protein's sequence alignment of +1 and -1 mutants; B and C, the resistance of dfrB3 and its frameshift mutants were evaluated on LB agar plate in the presence of TMP (final concentration=30μg/ml).

1.3.2. Purification and PAGE analysis of frame-shifted protein

To study the property of frame-shifted protein, the **+1 fs** (PK) protein was purified by affinity chromatography. Briefly, BL21(DE3) transformed by recombinant vectors was grown overnight and diluted 1:200 in fresh medium to OD600 0.4 in LB (250 rpm at 37 ºC). At this point, the temperature was lowered to 24 ºC and IPTG (20 μM) was added to induce the expression of DfrB3. The cells were then recovered by centrifugation (8000 rpm at 16 ºC for 15 min) and resuspended in 50mM Tris-HCl buffer (pH 7.9) containing 500mM NaCl and 10mM imidazole supplemented by 1mM phenylmethanesulphonylfluoride (PMSF). After mechanical lysis by sonication in an ice-cold bath, the soluble lysate was recovered by centrifugation (8000 rpm at 4 ºC for 30 min). Then the soluble fraction was filtered (0.45 μm) and loaded on His column according to the manufactory's manual. After the loaded column was washed with 4 column volumes (CV) of wash buffer (50mM Tris-HCl buffer pH 7.9 containing 500mM NaCl and 50mM imidazole). Finally, the proteins were eluted with 10mL of elution buffer (50mM Tris-HCl buffer pH 7.9 containing 500mM NaCl and 500mM imidazole) and the imidazole was removed by dialysis membrane.

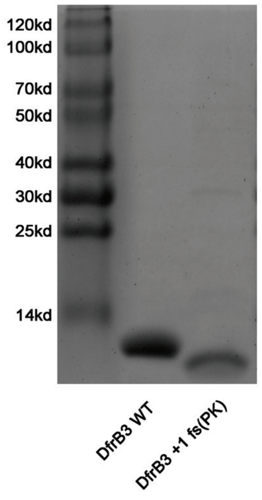

Figure S15. PAGE analysis of purified protein of DfrB3 WT and +1 fs(PK). Affinity chromatography purified proteins were analyzed by 12% SDS-PAGE.

The concentration of purified proteins was measured using nanodrop, and proteins were analyzed with 12% polyacrylamide gel electrophoresis (SDS-PAGE). Purified +1 fs(PK) proteins presented clear single band less than 14kD on SDS PAGE gel. But the band site of +1 fs(PK) was not same as WT protein (Figure S15).

1.3.3. Molecular weight determination by ESI-TOF MS

To study the amino acid composition of DfrB3, +1 fs(PK) and −1 fs(GG) mutant, the purified proteins were measured using electrospray ionization time-of-flight mass spectrometry (ESI-TOF MS): Agilent 1290 Infinity II LC systems-6545. Light sources used were a ZF-5A 16W 365nm UV source.

DfrB3, +1 fs(PK) and −1 fs(GG) mutant displayed specific peak as expected (Figure S16-S18). But the peak sites of three proteins were totally different (Figure S16-S18).

These results suggested that DfrB3, +1 fs(PK) and −1 fs(GG) were three proteins composed of different amino acid sequences.

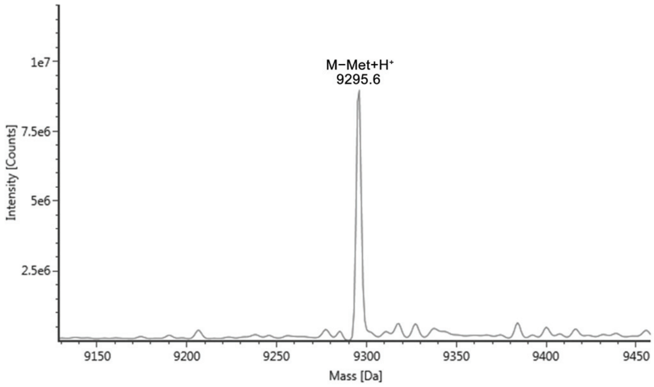

Figure S16. Molecular of DfrB3: protein was analysis by ESI-TOF MS. Observed peak at, expected peak at. The expected (M-Met+H^+) = 9296.3 Da. A peak with a mass of 9295.6 Da was measured.

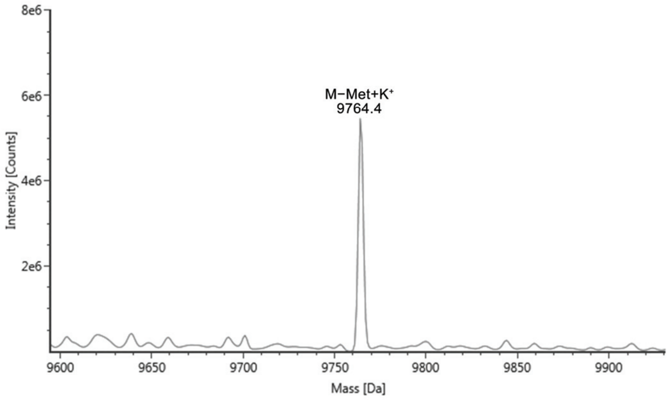

Figure S17. Molecular of +1 fs(PK): protein was analysis by ESI-TOF MS. The expected (M-Met+K^+) = 9758.4 Da. A peak with a mass of 9764.4 Da was measured.

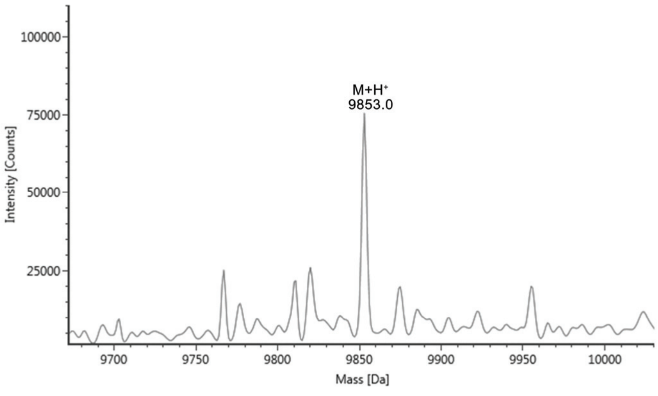

Figure S18. Molecular of −1 fs(GG): protein was analysis by ESI-TOF MS. The expected (M+H^+) = 9852.2 Da. A peak with a mass of 9853.0 Da was measured.

1.3.4. Analysis of AAR compositions of protein by LC-MS/MS

To further confirm the amino acid sequence of DfrB3, +1 fs(PK) and −1 fs(GG) mutant, the purified proteins were analyzed by ESI-TOF MS. Protein was digested by trypsin and molecular ion fragments were analyzed by ESI-TOF MS. LC-MS analysis was carried out using a Waters UPLC-MS (ESI), UPLC: Waters Acquity UPLC, MS: Waters Xevo G2-XS QTof. Purified proteins were digested by trypsin in 100mM NH4HCO3 at 37 ºC fir 12h, and filtered by using a 10 kDa Ultrafiltration centrifugal tube following desalinating and freeze-drying for preservation. The sample powders were re-dissolved in 15ul 0.1% FA, then centrifuged in 15000g for 15min. Supernatant was analyzed by LC-MS/MS.

+1 fs(PK) and −1 fs(GG) showed respective special molecular ion peak as expected (Figure S19 and S20). These results revealed that DfrB3, +1 fs(PK) and −1 fs(GG) were three proteins composed of different amino acid sequences.

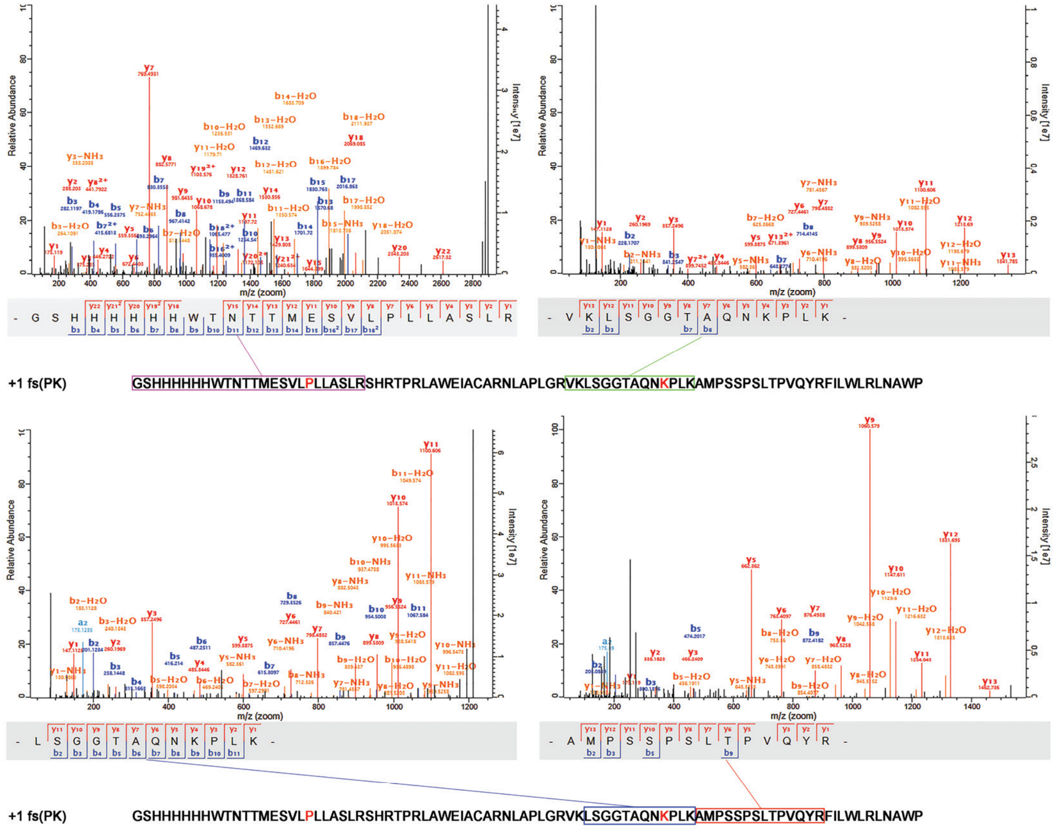

Figure S19. Tandem MS of +1 fs(PK)

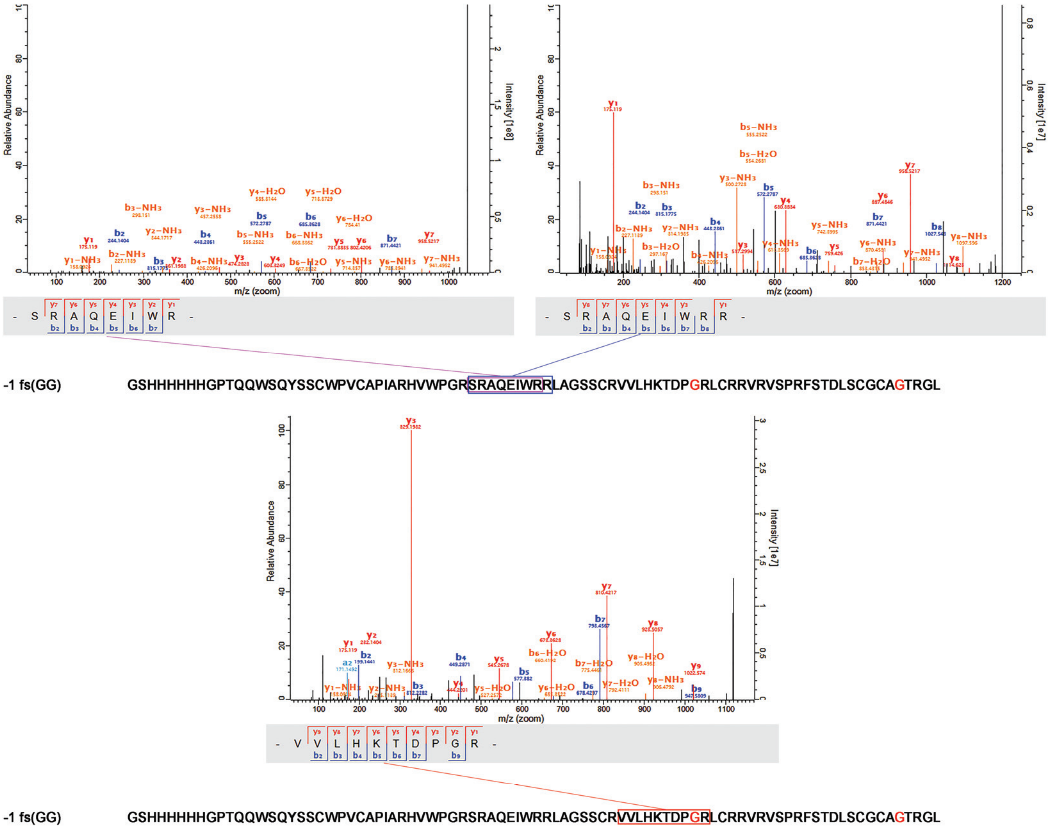

Figure S20. Tandem MS of −1 fs(GG)

1.3.5. Catalytic products analysis by UPLC-MS

WT DfrB3 reduces dihydrofolate (DHFA) into tetrahydrofolate (THFA) using NADPH as co-enzyme. To test the catalytic mechanism, UPLC-MS analysis was used to detect tetrahydrofolate production in the presence of NADPH. Reduction reactions were performed in the presence of DHFA (50 μM), NADPH (100 μM), and *dfrB3* proteins (6μg WT, or 6μg **+1 fs** (PK), or 12μg **-1 fs** (GG)) in 50 mM Tris buffer (pH 7.0) and 10 mM β-mercaptoethanol. And 6μg BSA was applied as the negative control to catalyze the same reduction reaction. All reactions were carried out for 60 minutes at 37 ºC, and then stopped by equal volume of methyl alcohol. The reaction mixture was centrifuged at 12000rpm for 10 min, and 1 μL of the supernatant was analyzed by UPLC-MS, using a Waters Vion IMS QTof coupled to a Waters Acquity UPLC system. A C18 reverse phase column was held at 40 ºC and a solvent system of aqueous formic acid 0.1% (v/v) (A) and acetonitrile (B) delivered at a flow rate of 0.2 mL/min. The gradient elution was applied as follows: 0–6 min, 5–25% B; 6–7 min, 25–95% B; 7–10 min, re-equilibration to initial conditions. The reactions and analysis were performed in duplicate. All processing was performed using MassLynx version 4.1. All data were acquired and analyzed by using Masslynx 4.1 software (Waters Corp., Beverly, MA).

After incubation of DHFA and NADPH WT DfrB3, +1 fs(PK) or −1 fs(GG), peak corresponding to THFA was be observed clearly by UPLC and MS, while control protein (BSA) could not produce THFA peak (Figure S21 to S24). Product of dehydrogenation, THFA, was detected by ESI-MS (Figure S21 to S24). All the results revealed that the frame-shifted +1 fs(PK) and −1 fs(GG) proteins catalyze same reduction reaction as WT Dfrb3.

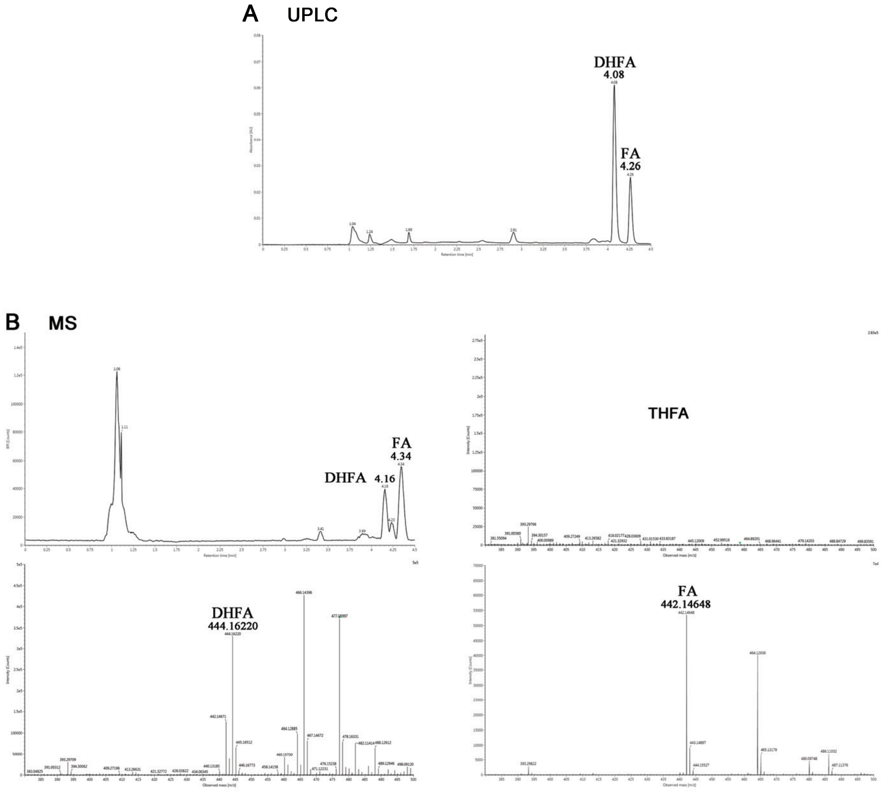

Figure S21. UPLC–MS/MS chromatogram of products catalyzed by BSA: A, UPLC spectrum of products catalyzed by BSA; B, MS chromatogram of products catalyzed by BSA, extracted mass spectrum of the peaks identified as THFA, DHFA and FA.

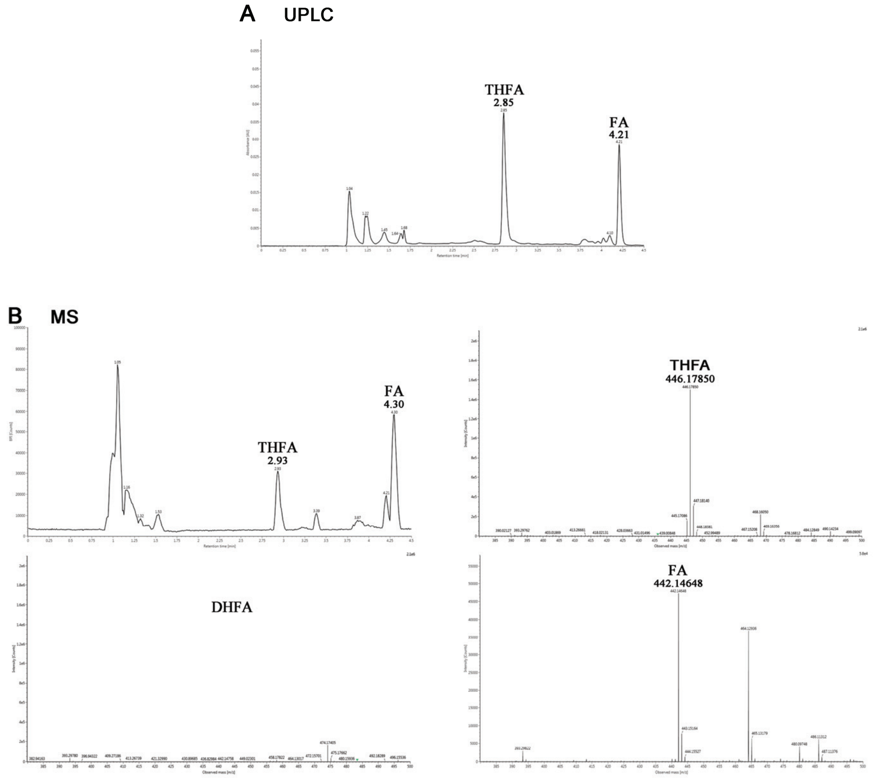

Figure S22. UPLC–MS/MS chromatogram of products catalyzed by DfrB3: A, UPLC spectrum of products catalyzed by DfrB3; B, MS chromatogram of products catalyzed by DfrB3, extracted mass spectrum of the peaks identified as THFA, DHFA and FA.

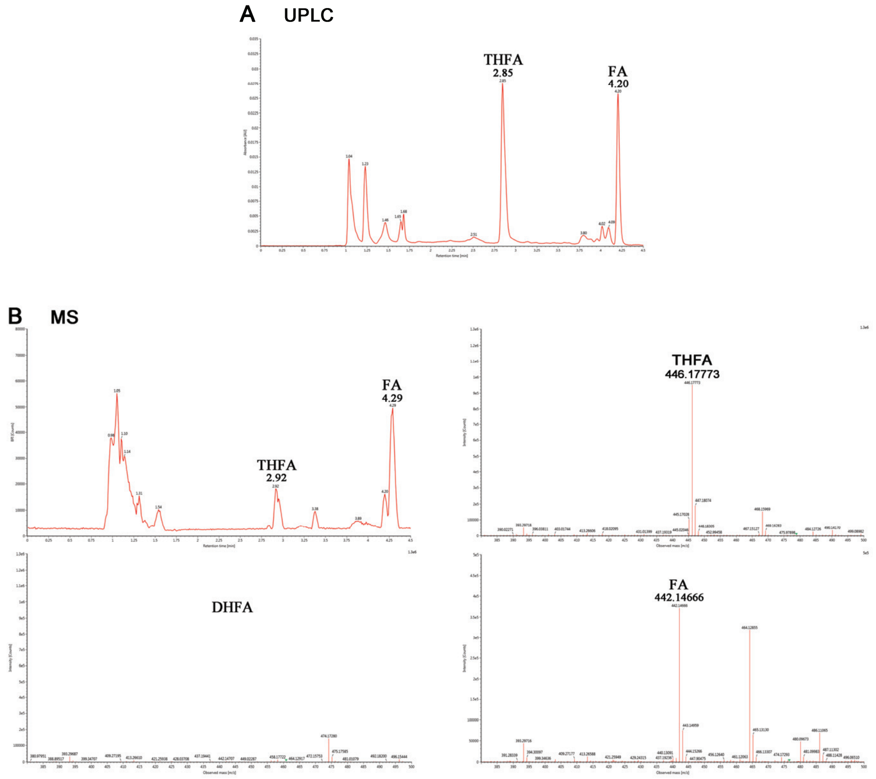

Figure S23. UPLC–MS/MS chromatogram of products catalyzed by DfrB3 +1 fs(PK): A, UPLC spectrum of products catalyzed by DfrB3 +1 fs(PK); B, MS chromatogram of products catalyzed by DfrB3 +1 fs(PK) , extracted mass spectrum of the peaks identified as THFA, DHFA and FA.

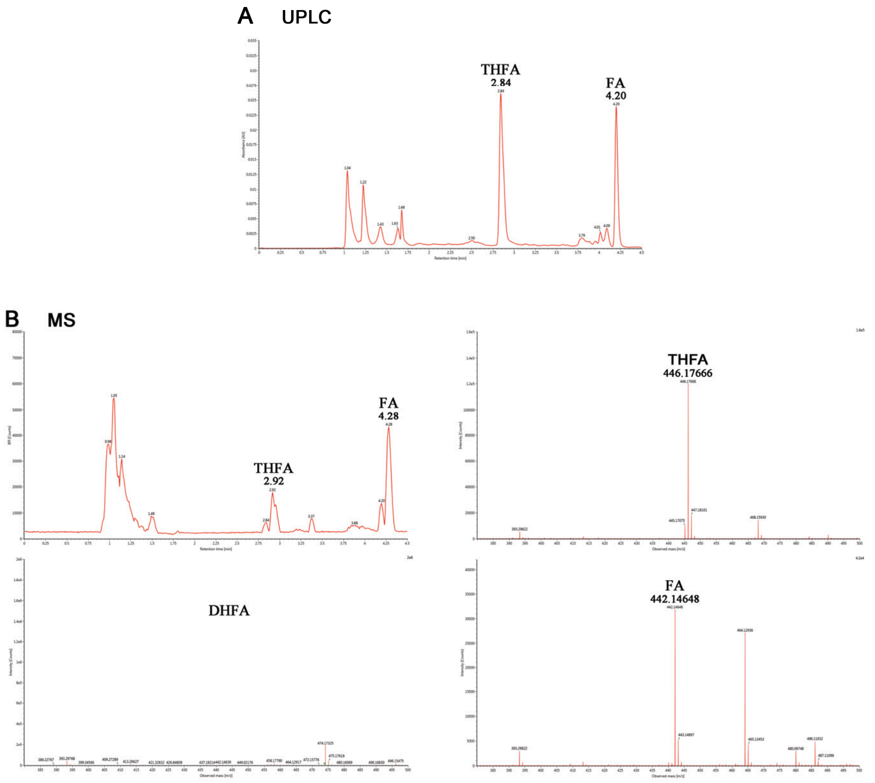

Figure S24. UPLC–MS/MS chromatogram of products catalyzed by DfrB3 −1 fs(GG): A, UPLC spectrum of products catalyzed by DfrB3 −1 fs(GG); B, MS chromatogram spectrum of products catalyzed by DfrB3 −1 fs(GG), extracted mass spectrum of the peaks identified as THFA, DHFA and FA.

1.3.6. Fluorescent tracking of the reduction reactions catalyzed by DfrB3, +1 fs (PK) and −1 fs(GG)

WT DfrB3 protein utilizes NADPH as the reductant to reduce dihydrofolate, yielding NADP as by-product. Because NADPH differ from NADP in fluorescence properties, the reduction reactions containing dihydrofolate and NADPH could be traced by measuring the fluorescent intensity at 460 nm. The reduction reaction was monitored using Thermo Scientific Microplate Reader by following the florescence decrease at 460 nm in the presence of DHFA (50 μM), NADPH (100 μM), and proteins (6μg BSA or 6μg DfrB3 or 6μg +1 fs(PK) or 12μg −1 fs(GG)) in 50 mM Tris buffer (pH 7.0) and 10 mM β-mercaptoethanol.

The control protein (BSA) could not cause obvious fluorescent change of reaction mixture containing dihydrofolate and NADPH, whereas both WT, +1 fs (PK) and −1 fs(GG) proteins of *dfrB3* caused the sharp decrease of fluorescence of the reaction system, indicating that the NADPH was being consumed rapidly (Figure S25).

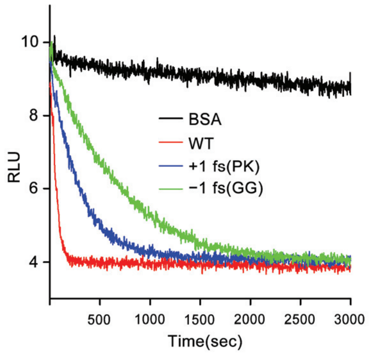

Figure S25. Fluorescent tracking of the reduction reactions catalyzed by BSA, WT, +1 fs (PK) and −1 fs(GG) of *dfrB3* at 460 nm.

1.3.7. Kinetic characterization of DfrB3, +1 fs (PK) and -1 fs (GG)

To further compare the catalytic rate quantitatively, the K_m, V_{max} and k_{cat} of three proteins were detected. Kinetic measurements were carried out as previous described (2). Briefly, kinetics was monitored in 5-80 μM DHFA and saturated NADPH (100 μM). The change in initial rate with concentration was fit to the Michaelis-Menten equation using GraphPad Prism 8. As showed in Figure S26, the catalytic rate of +1 fs(PK) and −1 fs(GG) is lower than that of WT. +1 fs (PK) and −1 fs(GG) have about 1/2 and 1/5 of the catalytic efficiency of WT enzyme respectively.

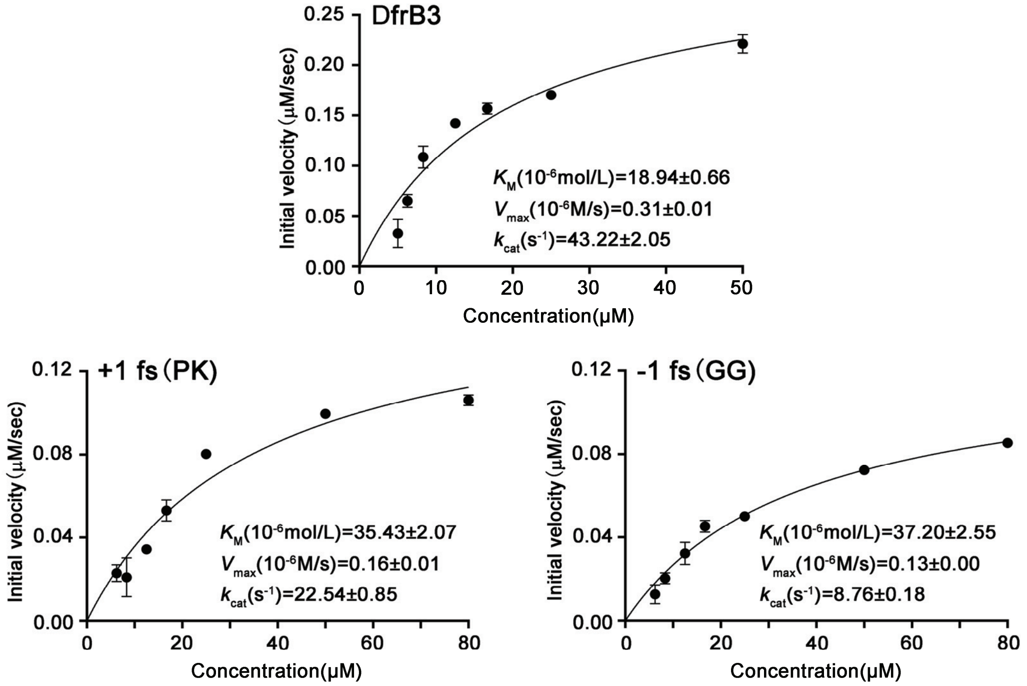

Figure S26. Kinetic characterization of WT, **+1 fs** (PK) and **-1 fs** (GG) of *dfrB3*.

2. Gene and protein sequences used in this study

2.1. Gene sequences

ibsC

ATGATGCGACTTGTCATCATACTGATTGTACTGTTACTCATAAGTTTCAGCGCTTATTAA

dinQ

ATGATTGATAAAGCAATCATCGTTCTAGGGGCGTTAATTGCGCTGCTGGAACTGATCCGCTTTCTGCT TCAGCTTCTGAACTGA

tisB

ATGAACCTGGTGGATATCGCCATTCTTATCCTCAAACTCATTGTTGCAGCACTGCAACTGCTTGATGC TGTTCTGAAATACCTGAAGTAA

ldrD

ATGACGTTCGCAGAGCTGGGCATGGCCTTCTGGCATGATTTAGCGGCTCCGGTCATTGCTGGCATTC TTGCCAGTATGATCGTGAACTGGCTGAACAAGCGGAAGTAA

pndA

ATGCCACAGCGAACGTTTTTAATGATGTTAATCGTCATCTGTGTGACGATTCTGTGTTTTGTCTGGAT GGTGAGGGATTCGCTTTGCGGACTCCGGCTCCAGCAGGGAAACACAGTGCTTGTGGCAACGTTAGCC TACGAAGTTAAACGTTAA

flmA

ATGAAACTACCACGCAGCTCTCTTGTCTGGTGTGTGTTGATCGTGTGTCTCACACTGTTGATATTCAC TTATCTGACACGAAAATCGCTGTGCGAGATTCGTTACAGAGACGGATACAGGGAGGTGGCGGCTTTCA TGGCTTACGAATCCGGTAAGTAG

GhoT

ATGGCACTATTCTCTAAAATATTAATTTTTTATGTGATTGGTGTGAACATATCCTTTGTCATTATCTGGT TTATCTCACATGAGAAAACACATATTCGTTTACTTAGTGCATTCCTGGTCGGAATAACCTGGCCAATGA GTCTGCCTGTGGCATTACTTTTTTCTCTCTTTTAG

dfrB3

ATGGACCAACACAACAATGGAGTCAGTACTCTAGTTGCTGGCCAGTTTGCGCTCCCATCGCACGCC ACGTTTGGCCTGGGAGATCGCGTGCGCAAGAAATCTGGCGCCGCTTGGCAGGGTCAAGTTGTCGGGT GGTACTGCACAAAACTGACCCCTGAAGGCTATGCCGTCGAGTCCGAGTCTCACCCCGGTTCAGTACAG ATTTATCCTGTGGCTGCGCTTGAACGCGTGGCCTGA

Full length toxin genes were prepared by fusion PCR using 2-4 primers as shown in Supplementary Table S1.

Full length gene of dfrB3 was chemical synthesized in Sangon Biotech (Sangon Biotech, Shanghai, China) for subclone to expression vectors pET-32.

2.2. Protein sequences

IbsC	MMRLVIILIVLLLISFSAY
DinQ	MIDKAIIVLGALIALLELIRFLLQLLN
TisB	MNLVDIAILILKLIVAALQLLDAVLKYLK
LdrD	MTFAELGMAFWHDLAAPVIAGILASMIVNWLNKRK
PndA	MPQRTFLMMLIVICVTILCFVWMVRDSLCGLRLQQGNTVLVATLAYEVKR
FlmA	MKLPRSSLVWCVLIVCLTLLIFTYLTRKSLCEIRYRDGYREVAAFMAYESGK
GhoT	MALFSKILIFYVIGVNISFVIIWFISHEKTHIRLLSAFLVGITWPMSLPVALLFSLF
DfrB3	MDQHNNGVSTLVAGQFALPSHATFGLGDRVRKKSGAAWQGQVVGWYCTKLTPEGYAVESES HPGSVQIYPVAALERVA

3. Supplementary Table

3.1. Supplementary Table S1. Primers of plasmid construction

Primer	Sequence
ibsC-F1	GTCGGATCCATGATGCGACTTGTCATCATACTGATTGTACTGTTACTCA
ibsC +1F1	GTCGGATCCTGATGCGACTTGTCATCATACTGATTGTACTGTTACTCA
ibsC +1F1n	GTCGGATCCnnnTGCGACTTGTCATCATACnnnTACTGTTACTCA
ibsC -1F1	GTCGGATCCGATGCGACTTGTCATCATACTGATTGTACTGTTACTCA
ibsC-3B	GCTGCTCAGCTTAATAAGCGCTGAAACTTATGAGTAACAGTACAA
dinQ-F1	GTCGGATCCATGATTGATAAAGCAATCATCGTTCTAGGGGCGTTAATTGCGCTGCTGGA
dinQ +1F1	GTCGGATCCTGATTGATAAAGCAATCATCGTTCTAGGGGCGTTAATTGCGCTGCTGGA
dinQ -1F1	GTCGGATCCGATTGATAAAGCAATCATCGTTCTAGGGGCGTTAATTGCGCTGCTGGA
dinQ-R2	GCTGCTCAGCTCAGTTCAGAAGCTGAAGCAGAAAGCGGATCAGTTCCAGCAGCGCAATT
tisB-F1	GTCGGATCCATGAACCTGGTGGATATCGCCATTCTTATCCTCAAACTCATTGTTGCAGC
tisB +1F1	GTCGGATCCTGAACCTGGTGGATATCGCCATTCTTATCCTCAAACTCATTGTTGCAGC
tisB -1F1	GTCGGATCCGAACCTGGTGGATATCGCCATTCTTATCCTCAAACTCATTGTTGCAGC
tisB-F2	CTCATTGTTGCAGCACTGCAACTGCTTGATGCTGTTCTG
tisB-R3	GCTGCTCAGCTTACTTCAGGTATTTCAGAACAGCATCAAG
ldrD-F1	GTCGGATCCATGACGTTCGCAGAGCTGGGCATGGCCTTCTGGCATGATTTAGCGGCTCC
ldrD +1F1	GTCGGATCCTGACGTTCGCAGAGCTGGGCATGGCCTTCTGGCATGATTTAGCGGCTCC
ldrD -1F1	GTCGGATCCGACGTTCGCAGAGCTGGGCATGGCCTTCTGGCATGATTTAGCGGCTCC
ldrD-F2	ATGATTTAGCGGCTCCGGTCATTGCTGGCATTCTTGCCAGTATGATCGTG
ldrD-R3	GCTGCTCAGCTTACTTCCGCTTGTTCAGCCAGTTCACGATCATACTGGCA
PndA-F1	GTCGGATCCATGCCACAGCGAACGTTTTTAATGATGTTAATCGTCATCTGTGTGACGAT
PndA +1F1	GTCGGATCCTGCCACAGCGAACGTTTTTAATGATGTTAATCGTCATCTGTGTGACGAT
PndA -1F1	GTCGGATCCGCCACAGCGAACGTTTTTAATGATGTTAATCGTCATCTGTGTGACGAT
PndAF2	CATCTGTGTGACGATTCTGTGTTTTGTCTGGATGGTGAGGGATTCGCTTTGCGGACTC
PndAR3	CGTAGGCTAACGTTGCCACAAGCACTGTGTTTCCCTGCTGGAGCCGGAGTCCGCAAAGC
PndAR4	GCTGCTCAGCTTAACGTTTAACTTCGTAGGCTAACGTTGC
FlmA-F1	GTCGGATCCATGAAACTACCACGCAGCTCTCTTGTCTGGTGTGTGTTGATCGTGTGTCT
FlmA +1F1	GTCGGATCCTGAAACTACCACGCAGCTCTCTTGTCTGGTGTGTGTTGATCGTGTGTCT
FlmA -1F1	GTCGGATCCGAAACTACCACGCAGCTCTCTTGTCTGGTGTGTGTTGATCGTGTGTCT
FlmA-F2	GTTGATCGTGTGTCTCACACTGTTGATATTCACTTATCTGACACGAAAATCGCTGTGCG

FlmA-R3	CATGAAAGCCGCCACCTCCCTGTATCCGTCTCTGTAACGAATCTCGCACAGCGATTTC
FlmA-R4	GCTGCTCAGCCTACTTACCGGATTCGTAAGCCATGAAAGCCGCCAC
GhoTS-F1	GTCGGATCCATGGCACTATTCTCTAAAATATTAATTTTTTATGTGATTGGTGTGAACAT
GhoTS +1F1	GTCGGATCCTGGCACTATTCTCTAAAATATTAATTTTTTATGTGATTGGTGTGAACAT
GhoTS -1F1	GTCGGATCCGGCACTATTCTCTAAAATATTAATTTTTTATGTGATTGGTGTGAACAT
GhoTS-F2	GATTGGTGTGAACATATCCTTTGTCATTATCTGGTTTATCTCACATGAGAAAACACATA
GhoTS-R3	ATTGGCCAGGTTATTCCGACCAGGAATGCACTAAGTAAACGAATATGTGTTTTCTCATG
GhoTS-R4	GCTGCTCAGCCTAAAAGAGAGAAAAAAGTAATGCCACAGGCAGACTCATTGGCCAGGTT
dfrB3-F	GTCGGATCCCACCACCACCACCACCACATGGACCAACACAACAATGGAG
dfrB3-R	GCTGCTCAGCTCAGGCCACGCGTTCAAGCGC
dfrB3 +1F	GTCGGATCCCACCACCACCACCACCACTGGACCAACACAACAATGGAG
dfrB3 +1R	GCTGCTCAGCTTAAGGCCACGCGTTCAAGCGC
dfrB3 +1 11Fn	GAGTCAGTACTCnnnTTGCTGGCCAGTTTG
dfrB3 +1 11Rn	CAAACTGGCCAGCAAnnnGAGTACTGACTC
dfrB3 +1 50Fn	GTACTGCACAAAACnnnCCCCTGAAGGCTATG
dfrB3 +1 50Rn	CATAGCCTTCAGGGGnnnGTTTTGTGCAGTAC
dfrB3 -1F	GTCGGATCCCACCACCACCACCACCACGGACCAACACAACAATGGAG
dfrB3 -1R	GCTGCTCAGCTTACAGGCCACGCGTTCAAGCGC
dfrB3 -1 52Fn	CACAAAACTGACCCCnnnAGGCTATGCCGTCG
dfrB3 -1 52Rn	CGACGGCATAGCCTnnnGGGGTCAGTTTTGTG
dfrB3 -1 74Fn	CCTGTGGCTGCGCTnnnACGCGTGGCCTG
dfrB3 -1 74Rn	CAGGCCACGCGTnnnAGCGCAGCCACAGG

3.2. Supplementary Table S2. Protein sequences of WT proteins and its frameshift mutations

Protein	Sequence
IbsC	
WT	MMRLVIILIVLLLISFSAY
+1 fs	-CDLSSY-LYCYS-VSALI…
−1 fs	DATCHHTDCTVTHKFQRLL…
DinQ	
WT	MIDKAIIVLGALIALLELIRFLLQLLN
+1 fs	-LIKQSSF-GR-LRCWN-SAFCFSF-T…
−1 fs	D--SNHRSRGVNCAAGTDPLSASASEL…
TisB	
WT	MNLVDIAILILKLIVAALQLLDAVLKYLK
+1 fs	-TWWISPFLSSNSLLQHCNCLMLF-NT-S…
−1 fs	EPGGYRHSYPQTHCCSTATA-CCSEIPEV…

LdrD		
	WT	MTFAELGMAFWHDLAAPVIAGILASMIVNWLNKRK
	+1 fs	-RSQSWAWPSGMI-RLRSLLAFLPV-S-TG-TSGS…
	−1 fs	DVRRAGHGLLA-FSGSGHCWHSCQYDRELAEQAEV…
PndA		
	WT	MPQRTFLMMLIVICVTILCFVWMVRDSLCGLRLQQGNTVLVATLAYEVKR
	+1 fs	CHSERF--C-SSSV-RFCVLSGW-GIRFADSGSSRETQCLWQR-PTKLN…
	−1 fs	ATANVFNDVNRHLCDDSVFCLDGEGFALRTPAPAGKHSACGNVSLRS-TL…
FlmA		
	WT	MKLPRSSLVWCVLIVCLTLLIFTYLTRKSLCEIRYRDGYREVAAFMAYESGK
	+1 fs	-NYHAALLSGVC-SCVSHC-YSLI-HENRCARFVTETDTGRWRLSWLTNPVS…
	−1 fs	ETTTQLSCLVCVDRVSHTVDIHLSDTKIAVRDSLQRRIQGGGGFHGLRIR-V…
GhoT		
	WT	MALFSKILIFYVIGVNISFVIIWFISHEKTHIRLLSAFLVGITWPMSLPVALLFSLF
	+1 fs	WHYSLKY-FFM-LV-TYPLSLSGLSHMRKHIFVYLVHSWSE-PGQ-VCLWHYFFLSF…
	−1 fs	GTIL-NINFLCDWCEHILCHYLVYLT-ENTYSFT-CIPGRNNLANESACGITFFSLL…
DfrB3		
	WT	MDQHNNGVSTLVAGQFALPSHATFGLGDRVRKKSGAAWQGQVVGWYCTKLTP<br>EGYAVESESHPGSVQIYPVAALERVA
	+1 fs	WTNTTMESVL-LLASLRSHRTPRLAWEIACARNLAPLGRVKLSGGTAQN-<br>PLKAMPSSPSLTPVQYRFILWLRLNAWP…
	−1 fs	GPTQQWSQYSSCWPVCAPIARHVWPGRSRAQEIWRRLAGSSCRVVLHKTDP-<br>RLCRRVRVSPRFSTDLSCGCA-TRGL…
**DfrB3**		**for purification**
	**WT**	MGSHHHHHHMDQHNNGVSTLVAGQFALPSHATFGLGDRVRKKSGAAWQGQVVGWYC<br>TKLTPEGYAVESESHPGSVQIYPVAALERVA Molecular weight=9426.5 Da
	**+1 fs(PK)**	MGSHHHHHHWTNTTMESVLPLLASLRSHRTPRLAWEIACARNLAPLGRVKLSGGTAQN<br>KPLKAMPSSPSLTPVQYRFILWLRLNAWP Molecular weight =9850.5 Da
	**−1 fs(GG)**	MGSHHHHHHGPTQQWSQYSSCWPVCAPIARHVWPGRSRAQEIWRRLAGSSCRVVLHK<br>TDPGRLCRRVRVSPRFSTDLSCGCAGTRGL Molecular weight =9851.2 Da

The substitution AAR was highlighted in red.

4. References

1. Mok, W.W., Patel, N.H. and Li, Y. (2010) Decoding toxicity: deducing the sequence requirements of IbsC, a type I toxin in Escherichia coli. *J Biol Chem*, **285**, 41627-41636.
2. Teixeira, B.V.F., Teles, A.L.B., Silva, S.G.D., Brito, C.C.B., Freitas, H.F., Pires, A.B.L., Froes, T.Q. and Castilho, M.S. (2019) Dual and selective inhibitors of pteridine reductase 1 (PTR1) and dihydrofolate reductase-thymidylate synthase (DHFR-TS) from Leishmania chagasi. *J Enzyme Inhib Med Chem*, **34**, 1439-1450.
